# Single‐cell atlas of peripheral blood mononuclear cells from pregnant women

**DOI:** 10.1002/ctm2.821

**Published:** 2022-05-06

**Authors:** Dongsheng Chen, Wei Wang, Linlin Wu, Langchao Liang, Shiyou Wang, Yunfeng Cheng, Tongda Zhang, Chaochao Chai, Qiuhong Luo, Chengcheng Sun, Wandong Zhao, Zhiyuan Lv, Ya Gao, Xiaoxia Wu, Ning Sun, Yiwei Zhang, Jing Zhang, Yixuan Chen, Jianing Tong, Xiangdong Wang, Yong Bai, Chaoyang Sun, Xin Jin, Jianmin Niu

**Affiliations:** ^1^ BGI‐Shenzhen Shenzhen China; ^2^ College of Life Sciences University of Chinese Academy of Sciences Beijing China; ^3^ Department of Obstetrics and Gynecology Tongji Hospital Tongji Medical College Huazhong University of Science and Technology Wuhan China; ^4^ Department of Obstetrics and Gynecology The Eighth Affiliated Hospital Sun Yat‐sen University Shenzhen China; ^5^ Jinshan Hospital Centre for Tumor Diagnosis and Therapy Fudan University Shanghai Medical College Shanghai China; ^6^ School of Basic Medicine Qingdao University Qingdao China; ^7^ Shenzhen Engineering Laboratory for Birth Defects Screening BGI‐Shenzhen Shenzhen China; ^8^ Department of Obstetrics Shenzhen Maternity and Child Healthcare Hospital Southern Medical University Shenzhen China; ^9^ Fudan University Shanghai Medical College Shanghai China; ^10^ School of Medicine South China University of Technology Guangzhou China

**Keywords:** interferon, PBMCs, pregnancy, scRNA‐seq

## Abstract

**Background:**

During pregnancy, mother–child interactions trigger a variety of subtle changes in the maternal body, which may be reflected in the status of peripheral blood mononuclear cells (PBMCs). Although these cells are easy to access and monitor, a PBMC atlas for pregnant women has not yet been constructed.

**Methods:**

We applied single‐cell RNA sequencing (scRNA‐seq) to profile 198,356 PBMCs derived from 136 pregnant women (gestation weeks 6 to 40) and a control cohort. We also used scRNA‐seq data to establish a transcriptomic clock and thereby predicted the gestational age of normal pregnancy.

**Results:**

We identified reconfiguration of the peripheral immune cell phenotype during pregnancy, including interferon‐stimulated gene upregulation, activation of RNA splicing‐related pathways and immune activity of cell subpopulations. We also developed a cell‐type‐specific model to predict gestational age of normal pregnancy.

**Conclusions:**

We constructed a single‐cell atlas of PBMCs in pregnant women spanning the entire gestation period, which should help improve our understanding of PBMC composition turnover in pregnant women.

## Graphical abstract

We generated a single‐cell atlas of PBMCs in pregnant women; Pregnancy activates interferon (IFN) responses versus non‐pregnant control in NK/T cells; T‐, B‐cell activities attenuated significantly during gestation; Cell‐type‐specific model accurately predicts gestational age.

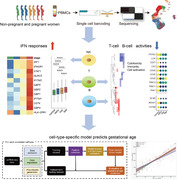

## 1 INTRODUCTION

During pregnancy, homeostasis of the maternal immune system is critical for pregnancy success, conferring tolerance to the semi‐allogenic fetus, while maintaining the ability to protect against pathogens.^1^, ^2^ Dysregulation of immunological mechanisms underlies various pregnancy‐related pathologies, such as preterm labour, preeclampsia and other complications.^3^, ^4^ Early capture of these dysregulated processes during pregnancy is highly desirable for risk prediction and mitigation. Therefore, there is a great need for a comprehensive understanding of the changes in immune features that occur throughout normal pregnancy.

The mechanisms that underpin fetomaternal immune adaptation during pregnancy have been extensively researched.^5^, ^6^, ^7^ However, most studies have explored immunomodulatory mechanisms at the local fetomaternal interface. Furthermore, due to ethical considerations, studies have focused on early pregnancy and postpartum, and therefore may not accurately reflect changes in the immune system throughout pregnancy. Moreover, fetomaternal cross‐talk not only influences local fetomaternal cellular mechanisms that control maternal immune tolerance to the semi‐allogeneic fetus, but also systemic immune adaptations to pregnancy.^8^ Growing evidence indicates that pregnancy is accompanied by alterations in the immune system in maternal systemic circulation,^9^ e.g., pro‐inflammatory activity of natural killer (NK) cells is upregulated in pregnancy,^10^ and frequencies of B cells are significantly downregulated during gestation.^9^

The application of cytomics and single‐cell transcriptomics has provided an unprecedented ability to capture the complexities of systemic immunological adaptations during pregnancy.^11^, ^12^ Recent research showed that chronological and predictable variations in immune features can be tracked in peripheral blood over the course of a full‐term pregnancy, and signal transducer and activator of transcription (STAT) 5 signalling in several CD4+ T‐cell subsets increases significantly as pregnancy progresses.^8^ A subsequent study revealed that disruption in STAT5 signalling dynamics in CD4+ T cells is highly correlated with later stage preeclampsia,^13^ suggesting that peripheral blood mononuclear cells (PBMCs) may potentially reflect normal or pathological pregnancy status. However, a detailed understanding of the cellular interactions and mechanisms in PBMCs over the course of gestation is lacking.

Here, we used single‐cell transcriptome analysis to profile 198,356 cells obtained from pregnant (gestation weeks 6–40) and non‐pregnant women, representing a comprehensive and systematic immunological signature of normal pregnancy. Additionally, we developed a cell‐type‐specific model to predict gestational age (in days) in normal pregnancy. By completing PBMC type annotation and identifying dynamic changes in PBMC abundance and molecular characteristics, we constructed a detailed cellular taxonomy of pregnant woman PBMCs spanning the entire gestational period.

## 2 RESULTS

### 2.1 Longitudinal analysis of PBMCs during pregnancy: unbiased and high‐density sampling

**FIGURE 1:**
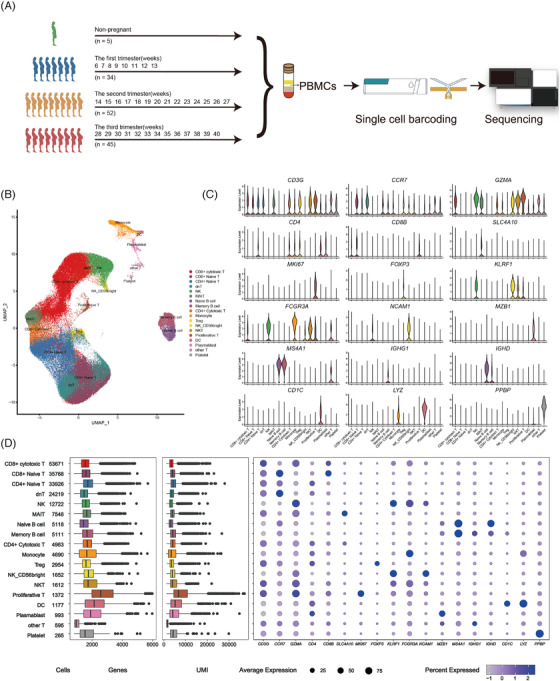
Single‐cell gene expression profiling of PBMCs of the pregnant and non‐pregnant women. (A) Schematic representation of the study design. (B) The clustering result of 198, 356 cells from 136 donors. Each point represents one single cell, coloured according to cell type. (C) Violin plots of expression values for cell type‐specific marker genes. (D) From left to right are the cell number of each cell type, the box plot of the number of genes, the box plot of unique molecular identifiers (UMI) and the bubble plot of cell type‐specific marker genes

To capture transcriptional dynamics of PBMCs during pregnancy, we collected 131 PBMC samples from gestational week 6 (GW6) to GW40 and five non‐pregnant samples as the control group (Figure 1A, Table S1). In total, 198,356 cells were retained after filtration, which were annotated into 18 main cell types, including mucosal associated invariant T cells (MAIT, CD3G+SLC4A10+), CD3+CD4–CD8– double negative T cells (dnT, CD3G+CCR7+), proliferative T cells (proliferative T, CD3G+MKI67+), CD4+ cytotoxic T cells (CD3G+CD4+GZMA+), CD4+ naïve T cells (CD3G+CD4+CCR7+), CD8+ naïve T cells (CD8A+CCR7+), CD8+ cytotoxic T cells (CD8A+GZMA+), other T cells (CD3G+), memory B cells (MS4A1+IGHG1+), naïve B cells (MS4A1+IGHG1‐IGHD+), plasmablasts (MZB1+XBP1+IRF4), CD56‐dim natural killer (NK) cells (CD3G‐KLRF1+FCGR3A+), CD56‐bright natural killer cells (NK_CD56‐bright cells, CD3G‐KLRF1+NCAM1+), monocytes (FCGR3A+LYZ+), dendritic cells (DCs, LYZ+CD1C+), Tregs (Regulatory T) (CD4+FOXP3+CTLA4+), NK T cells (NKT, CD3G+KLRF1+FCGR3A+) and platelets (PPBP+) (Figure 1B–1D, S1A, S1C). Most cell types consisted of cells from multiple samples, indicating common immune traits among pregnant and non‐pregnant women (Table S2). We identified significantly highly expressed genes in each cell type and performed functional enrichment analysis using Gene Ontology (GO). The top five most significantly enriched GO terms in each cell type were consistent with corresponding function (Figure S1B). Mature T cells showed activation in lymphocyte immune functions, including interleukin‐2 production and cell adhesion, while proliferative T cells were involved in synthesis and metabolism of adenosine triphosphate (ATP). The B cells were associated with activation of B‐cell receptor signalling. All NK cells and monocytes exhibited functional characteristics of immune response activation, while DCs were involved in pre‐processing and presentation of antigens. Platelets were involved in coagulation‐associated functions. These results demonstrated the reliability of our dataset.

### 2.2 Dynamics of immune cell abundance during pregnancy

**FIGURE 2:**
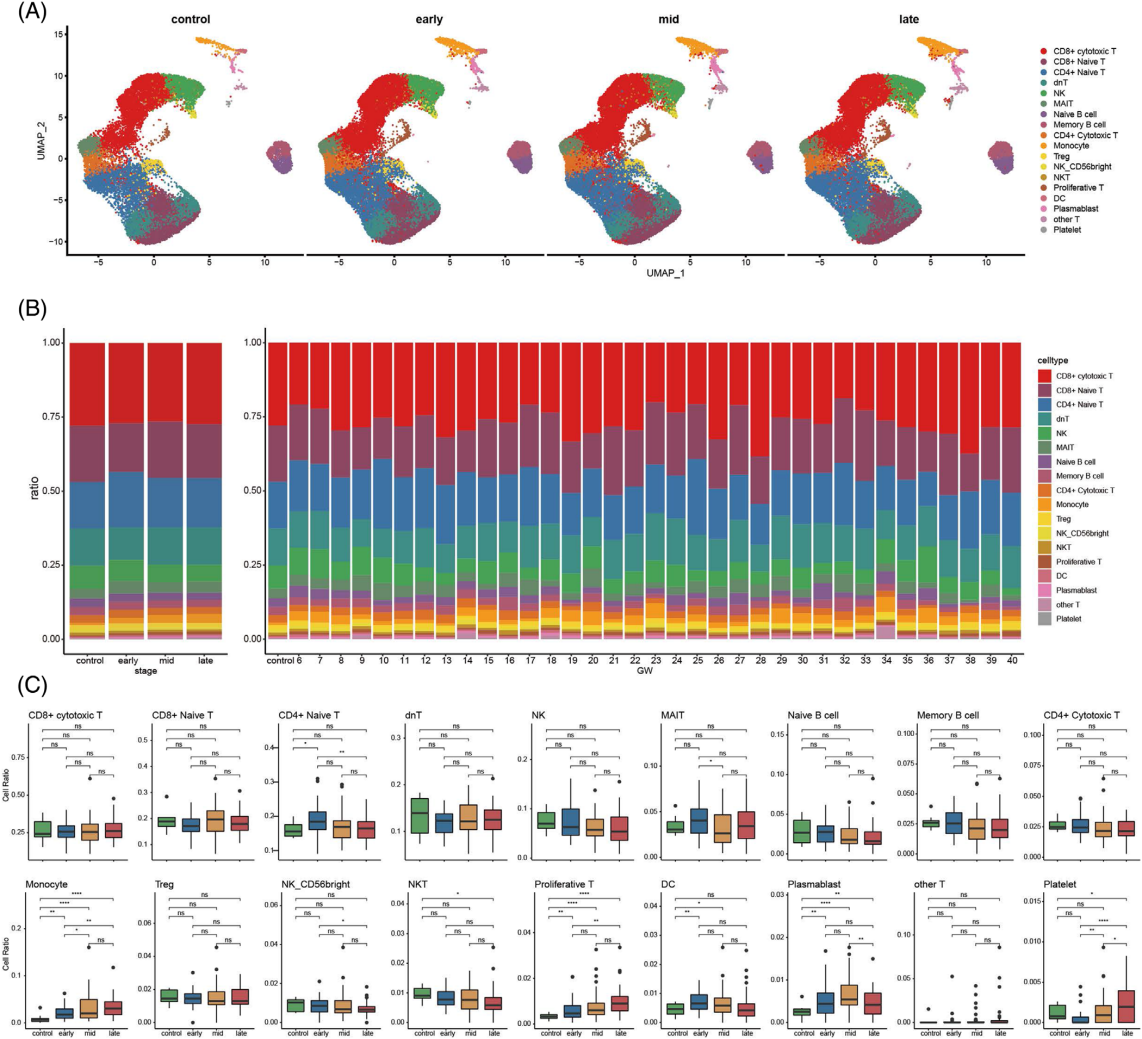
Differences in cell compositions by single‐cell transcriptomes of PBMCs during pregnancy. (A) UMAP plot of cell composition in different pregnancy period. (B) Proportion of each cell type at different pregnancy (left) and different GW (right). Bars are coloured by cell types. (C)The histogram shows the number of differentially expressed genes (DEGs) during three periods of pregnancy compared to control group. (upper panel: up‐regulated DEGs; lower panel: down‐regulated DEGs). (D) Box plot shows cell type proportion changes of each pregnancy period. Conditions are shown in different colours. Horizontal lines represent median values, with whiskers extending to the farthest data point within a maximum of 1.5 × interquartile range. Student's t test was applied. All differences with *p* < 0.05 are indicated. * *p* < 0.05, ** *p* < 0.01, *** *p* < 0.001,**** *p* < 0.0001, ns = not significant

All immune cells were divided into four stages based on gestational week (i.e., early‐, mid‐ and late‐pregnancy stage and non‐pregnant) (Figure 2A). To investigate whether the PBMC abundance changed during pregnancy, we calculated the percentages of all cell types in different pregnancy stages and different gestational weeks (Figure 2B). In general, there were no significant differences among the four stages of pregnancy in most immune cell types, except for monocytes, proliferative T cells and plasmablasts. Monocytes increased significantly during pregnancy, beginning at the first trimester (Figure 2C), consistent with previous research.^14^, ^15^, ^16^, ^17^ The increase in monocyte abundance is usually accompanied by an unresponsive state during pregnancy.^18^ Except for proliferative T cells, the percentages of most lymphatic cells did not differ significantly during pregnancy, as reported previously.^19^ Among these lymphatic cells, CD4+ naïve T cells showed a slight peak in the first trimester of pregnancy. Furthermore, proliferative T cells increased significantly during pregnancy compared to non‐pregnancy. It has been reported that almost all B‐cell subtypes decrease during pregnancy.^20^ Here, although naïve and memory B cells did not differ significantly during the four stages of pregnancy, there was a slight decrease from non‐pregnancy to late pregnancy. Plasmablasts, that is, short‐lived differentiation stage between post‐germinal centre B cells and mature plasma cells, increased progressively in the first and second trimester of pregnancy but decreased in the third trimester. However, the percentage of plasmablasts in the third trimester was higher than that during non‐pregnancy, contrary to previous reports.^21^

### 2.3 IFN responses enhanced in innate immune cells

**FIGURE 3:**
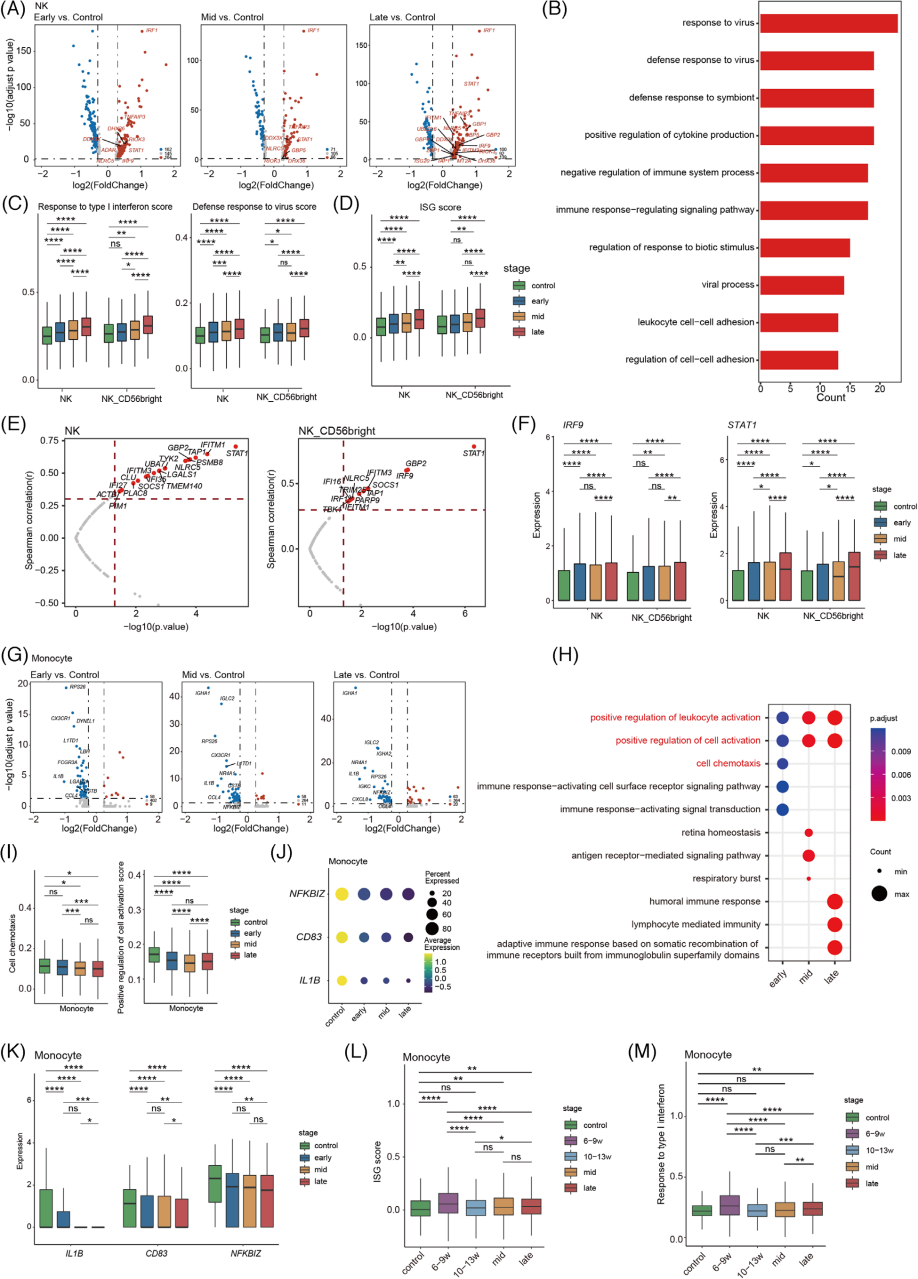
Dynamic functional changes in NK cells and monocytes of PBMCs during pregnancy. (A)Volcano plots of DEGs in all NK cells (NK and NK_CD56 bright cells). Results from left to right represent first trimester versus non‐pregnancy, second trimester versus non‐pregnancy and third trimester versus non‐pregnancy respectively. Red points represent upregulated genes, while blue points were downregulated genes in pregnant women. Genes with |log2(FC)| ≥ 0.3, adjusted *p* < 0.05 and related IFN were labelled by gene symbols. (B) GO term enrichment of genes which highly expressed in third trimester compared to non‐pregnancy. (C) Box plots of the expression levels of two GO biological process terms in NK/NK_CD56 bright cells derived from three periods of pregnancy and non‐pregnant samples. Wilcoxon rank‐sum test was applied. (D) Box plots of the collected ISGs scores across different clusters and conditions. Statistical significance of difference in pairs among four pregnant stages were labelled at the top of box plot. Wilcoxon rank‐sum test was applied. (E) Correlation test between ISGs expression level in NK/NK_CD56 bright cells with gestational weeks. Correlation analysis using the Pearson's product‐moment correlation. (F) Expression level of *STAT1* and *IRF9* in NK/NK_CD56 bright cells. Statistical significance of difference in pairs among four stages were labelled at the top of box plot. Wilcoxon rank‐sum test was applied. (G) Volcano plots of differentially expressed genes in monocytes, which the order is consistent with (A). (H) The top 5 significant GO terms enriched by genes highly expressed in three pregnant stages compared to non‐pregnancy. (I) Box plots of the cell scores for two GO biological process terms derived from early, mid, late and non‐pregnant control samples. Wilcoxon rank‐sum test was applied. (J) Dot plot of three representative low expressed genes (*IL1B*, *CD83* and *NFKBIZ*) in monocytes. Dots sizes represent the proportion of cells expressed in four stages. Dot colours represent average expression levels of monocytes in four stages. (K) Expression level of *IL1B*, *CD83* and *NFKBIZ* in monocytes. Statistical significance of difference in pairs among four stages was labelled at the top of box plot. (L) Box plots of the collected ISGs scores across different periods in monocyte. Wilcoxon rank‐sum test was applied. (M)Box plots of expression levels in GO biological process term of response to type I interferon in monocytes. Wilcoxon rank‐sum test was applied. All differences with *p* < 0.05 are indicated.* *p* < 0.05, ** *p* < 0.01, *** *p* < 0.001, **** *p* < 0.0001, ns = not significant

While it is known that the innate immune system is activated during pregnancy,^9^ previous studies have focused on invasive NK cells at the fetomaternal interface, with NK cells in peripheral blood more poorly understood. To resolve the dynamic changes in innate immune cells in maternal systemic circulation with progressing pregnancy, we examined differences in NK (NK and NK_CD56‐bright) cells among the three periods of pregnancy and the non‐pregnant state. Compared to non‐pregnancy, the upregulated DEGs in NK (NK and NK_CD56‐bright) cells were involved in biological processes, including IFN and virus responses, in all three periods of pregnancy (Figure 3A). GO analysis also showed that the upregulated genes in NK cells were enriched in defense response to virus, positive regulation of cytokine production during the third trimester (Figure 3B), suggesting that IFN responses, virus responses in NK cells are significantly enhanced during pregnancy. Furthermore, the expression of genes involved in responses to IFN and virus increased progressively with pregnancy, suggesting that responsiveness to IFN in NK/NK_CD56‐bright cells increases during pregnancy (Figure S2A–C). To estimate the expression level of gene sets in each cell, we binned features based on average expression and randomly selected 100 control features from each bin. Aggregated expression of gene set features was subtracted by the aggregated expression of the control feature set to obtain a gene set score. To demonstrate the dynamic patterns of responses to IFN and virus, we determined the activities of two important GO pathways (i.e., response to type I IFN and defense response to virus) in the NK/NK_CD56‐bright cells. As expected, the expression scores of the two immune responses increased significantly during pregnancy and tended to be higher with pregnancy progression, suggesting that enhanced responsiveness to IFN and virus is the result of global enhancement rather than limited to specific genes (Figure 3C). Moreover, in the NK/NK_CD56‐bright cells, 20 IFN‐stimulated genes (ISGs) were significantly positively correlated with gestational week (Figure 3E and S2D). *STAT1*, which mediates cellular response to IFNs,^22^, ^23^ showed the strongest correlation (NK: cor 0.67, *p* = 8.99e‐06; NK_CD56‐bright: cor 0.77, *p* = 7.35e‐08) (Figure 3E). *STAT1* can regulate ISG expression by forming the complex *ISGF3* in combination with *STAT2* and *IRF9*.^24^ In contrast, *STAT1* and *IRF9* increased progressively during pregnancy (Figure 3F). The ISG score based on collected ISGs also displayed a progressive increase during pregnancy (Figure 3D). These results suggest that *STAT1* may play an important role in activating the immune response to IFN in NK/NK_CD56‐bright cells during pregnancy.

We also identified DEGs in monocytes between pregnancy and non‐pregnancy (Figure 3G). Most significant DEGs decreased during pregnancy and were associated with cell chemokines and positive regulation of the cell activation signalling pathway (Figure 3H). DEGs involved in these two functions decreased significantly during pregnancy (Figure S2E and F). We further evaluated the expression levels of these two GO pathways (i.e., cell chemokines and positive regulation of cell activation). Compared to non‐pregnancy, the activity of both pathways decreased significantly in the three periods of pregnancy (Figure 3I). Among the genes associated with positive regulation of cell activation, *IL1B*, *CD83* and *NFKBIZ*, which are key regulators of immune cell activation,^25^, ^26^ decreased during pregnancy (Figure 3J and 3K). To evaluate response to IFN in the monocytes, we explored ISG expression patterns with pregnancy. Some ISGs (e.g., *AIM2*, *ZBP1*, *IRF7*, *PLSCR1* and *IFITM1*) displayed a conversion from the early to late stage in the first trimester of pregnancy (Figure S2G), indicating there may be a more complex response to IFN in monocytes during early pregnancy. Based on the ISG expression patterns in the monocytes in early pregnancy, we divided early pregnancy into early stage (GW6–9) and late stage (GW10–13). *IRF1* and *IFITM1* showed higher expression in the early stage than in the late stage (Figure &nbsp;S2H), while *LST1*, *MAFB* and *CEBPB* were higher in the late stage than in the early stage. *LST1* isoforms are associated with immunosuppression function.^27^ *MAFB*+ macrophages regulate tissue homeostasis and immunosuppression.^28^ *CEBPB* is involved in immunosuppression in cancer.^29^ Gene set enrichment analysis (GSEA) of DEGs showed that highly expressed genes in the early stage were enriched in IFN gamma response, while those in the late stage were involved in negative regulation of immune system process (Figure S2I and J). The ISG score based on all collected ISGs and immune score of response to type I IFN peaked in the early stage, then decreased significantly in the late stage (Figure 3L and 3M). These results suggest that immunosuppression increases during pregnancy, especially after GW9.

### 2.4 Features of T‐cell subsets during pregnancy

**FIGURE 4:**
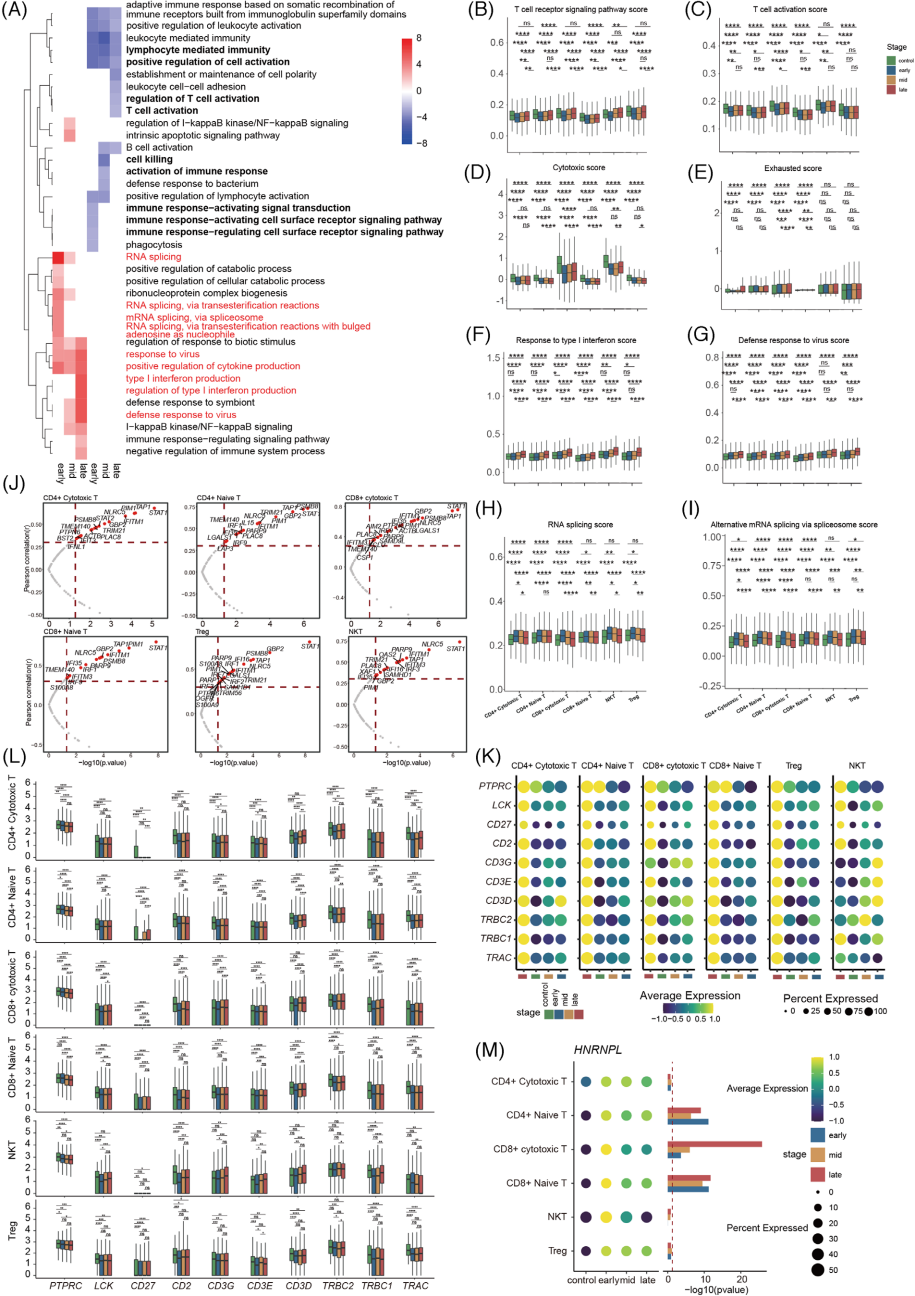
Features of T‐cell subsets during pregnancy. (A) GO term enrichment of genes which highly expressed in different trimester compared to non‐pregnancy in T cell (CD8+ naïve T, CD8+ cytotoxic T, CD4+ naïve T, CD4+ cytotoxic T, NKT, Treg, proliferative T, MAIT and other T). Red means upregulation compared to non‐pregnancy, blue means downregulation compared to non‐pregnancy. (B and C) Boxplots of the cell scores of two GO biological process terms (T‐cell receptor signalling pathway and T‐cell activation) in CD4+ T, CD8+ T, NKT and Treg cells across four conditions. Wilcoxon rank‐sum test was applied. (D and E) Box plots of the cell scores for CD4+ T, CD8+ T, NKT and Treg cells of cytotoxic and exhausted associated genes across four conditions. Wilcoxon rank‐sum test was applied. (F and G) Box plots of the cell scores of two GO biological process terms (response to type I interferon and defense response to virus) in CD4+ T, CD8+ T, NKT and Treg cells across four conditions. Wilcoxon rank‐sum test was applied. (H and I) Box plots of the cell scores of two GO biological process terms (RNA splicing and alternative mRNA splicing via spliceosome) in CD4+ T, CD8+ T, NKT and Treg cells across four conditions. Wilcoxon rank‐sum test was applied. (J) Correlation test between ISGs expression level in CD4+ T, CD8+ T, NKT and Treg cells with gestational weeks. Correlation analysis using the Pearson's product‐moment correlation. (K) Dot plot of ten genes expression pattern across four conditions, including *CD27*, *TRAC*, *TRBC1*, *TRBC2*, *CD3D*, *CD3E*, *CD3G*, *CD2*, *LCK* and *PTPRC*. (L) The difference in expression levels of above ten genes in CD4+ T, CD8+ T, NKT and Treg cells between three periods of pregnancy and non‐pregnant controls. (M) *HNRNPL* expression pattern across four conditions, the graph on the right shows the significance of three periods of pregnancy compared to non‐pregnant controls. Wilcoxon rank‐sum test was applied. All differences with *p* < 0.05 are indicated. * *p* < 0.05, ** *p* < 0.01, *** *p* < 0.001, **** *p* < 0.0001, ns = not significant

To further explore transcriptomic changes in T cells during pregnancy, we compared expression patterns in the first/second/third trimester with non‐pregnant control. Results showed that the downregulated DEGs were mainly involved in positive regulation of cell activation, T‐cell activation, immune response−activating signal transduction during pregnancy, while the upregulated DEGs primarily participated in IFN response, virus response, cytokine production and RNA splicing‐related pathways (Figure 4A, S3A and B). Moreover, as pregnancy progressed, the number of downregulated genes related to T‐cell activation and upregulated genes associated with the RNA splicing pathways decreased, whereas the upregulated genes involved in IFN and virus responses increased in the T cells (Figure S3A). To gain further insight into T‐cell activation status, RNA splicing and IFN response at various stages of pregnancy, we assessed the expression levels of six significant GO pathways (i.e., T‐cell receptor [TCR] signalling pathway, T‐cell activation, RNA splicing, alternative mRNA splicing via spliceosome, response to type I IFN and defense response to virus) in the T cells (CD4+ naïve T, CD4+ cytotoxic T, CD8+ naïve T, CD8+ cytotoxic T, NKT, Treg cells). Results showed that the TCR signalling pathway and T‐cell activation pathway scores were significantly downregulated in T cells during pregnancy. Moreover, early pregnancy had the lowest scores, whereas late pregnancy showed slightly higher scores in the CD4+ and CD8+ T cells (Figure 4B and C, S3C and D). Using the cytotoxic and exhausted scoring system, most T‐cell subsets had a lower cytotoxicity score but higher exhaustion score during pregnancy compared to pre‐pregnancy. In addition, the CD8+ cytotoxic T and NKT cells showed higher cytotoxicity scores than those of the other subsets. Within these highly cytotoxic clusters, the cytotoxicity score was slightly elevated from early to late gestation (Figure 4D and 4E, S3E and F). These results indicate that T‐cell activity is significantly attenuated throughout pregnancy but is mildly enhanced from early to late gestation.

IFN and virus responses were significantly increased in pregnancy relative to non‐pregnancy, and increased with pregnancy progress (Figure 4F and 4G, S3G‐I). We identified 28 ISGs that showed positive correlation with gestational weeks in T cells (Figure 4J, S3J). Previous research has shown that IFN contributes to T‐cell activation.^30^ However, our results suggested that T‐cell activity is attenuated, while IFN responses are enhanced during pregnancy. Effective T‐cell activation is achieved only when the responding T cells integrate three signals: that is, binding of an antigen to TCR (first signal), ligation of co‐stimulatory molecules (second signal) and activation of specific cytokine signals (third signal). IFNs were widely studied third signal for T cells.^30^ Hence, we compared the expression levels of associated receptors and molecules of T cells (CD4+ naïveT, CD4+ cytotoxic T, CD8+ naïve T, CD8+ cytotoxic T, NKT, Treg cells) between the first, second and third trimester and non‐pregnancy. We found that TCRs (*TRAC*, *TRBC1* and *TRBC2*), *CD3D*, *CD3E* and *CD3G* (proteins encoded by *CD3D*, *CD3E* and *CD3G* combine with TCRs to form a TCR‐CD3 complex, which promotes T‐cell activation^31^) and co‐stimulatory receptors (*CD2* and *CD27*)^32^, ^33^ were all significantly downregulated in T cells (CD4+ naïve T, CD4+ cytotoxic T, CD8+ naïve T, CD8+ cytotoxic T, Treg cells). Moreover, *LCK*, which encodes the first kinase transducing TCR signal critical for T‐cell development and activation,^34^ and *CD45*, a positive regulator of LCK,^35^ were also reduced in the T‐cell subsets during pregnancy (Figure 4K‐4L).

RNA splicing, especially alternative splicing, is critical in eukaryotic gene regulation. Alternative splicing plays an important role in maintaining T‐ and B‐cell homeostasis in the peripheral immune system.^36^, ^37^ Here, we observed that RNA splicing and alternative mRNA splicing via spliceosome pathways were significantly upregulated in the T‐cell subsets across pregnancy, although the pathway scores gradually decreased as pregnancy progressed (Figure 4H‐4I, S3K). Moreover, *HNRNPL*, which encodes a well‐characterised RNA‐binding protein involved in alternative splicing and plays an important role in regulating both TCR and BCR‐dependent activation,^37^, ^38^ was overexpressed in T cells (CD4+ naïve T, CD4+ cytotoxic T, CD8+ naïve T, CD8+ cytotoxic T, NKT, Treg cells) throughout pregnancy, especially in early gestation (Figure 4M). These results suggest that alternative mRNA splicing may play an important role in regulating T‐cell activity during pregnancy.

Collectively, our findings indicated that T‐cell activity decreased throughout pregnancy, possibly due to first and second signal deficiencies and upregulation of alternative mRNA splicing. Furthermore, the limited enhancement in T‐cell activity from early to late pregnancy may be due, in part, to the upregulation of IFN responses during pregnancy.

### 2.5 Attenuation of B‐cell activity during gestation

**FIGURE 5:**
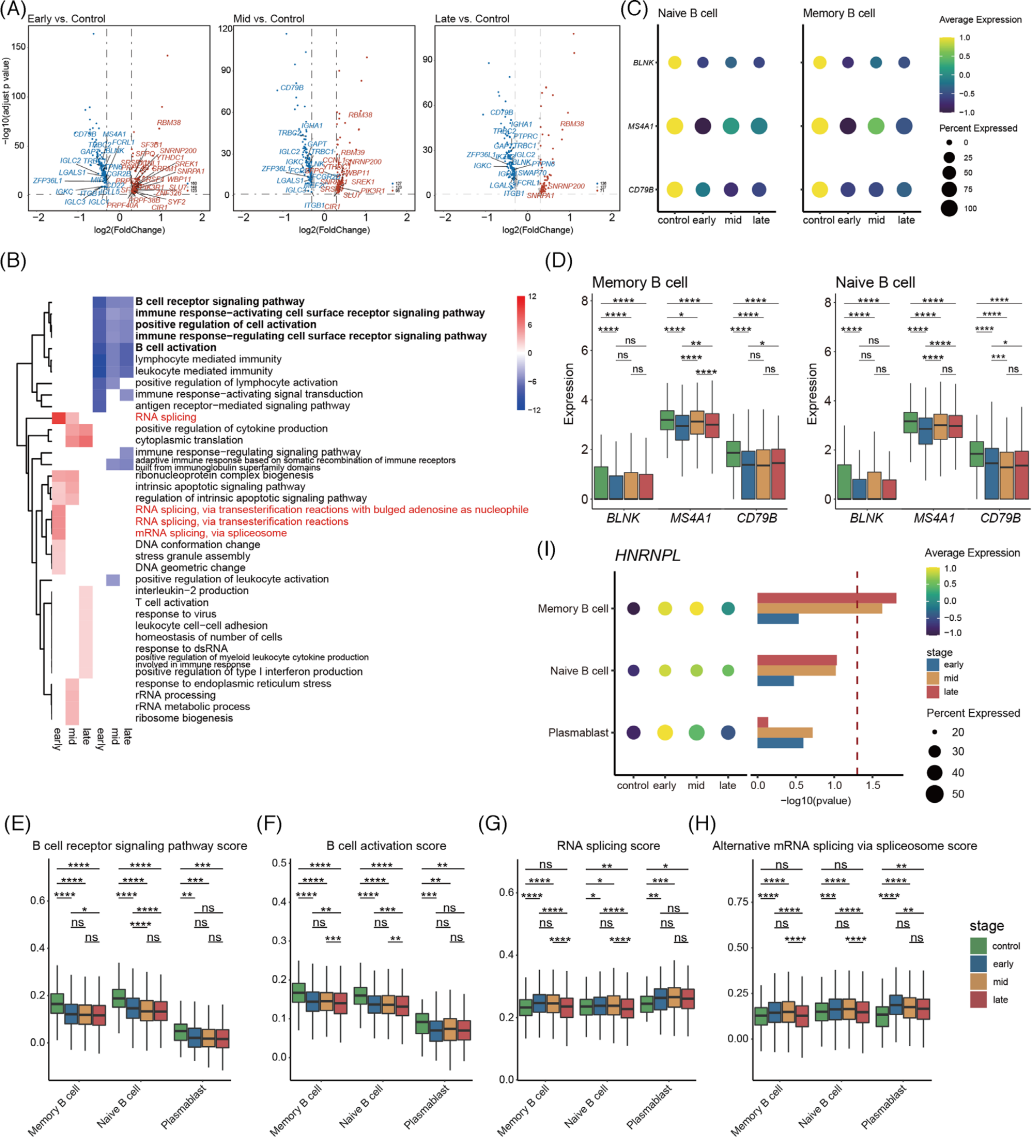
Dynamic functional changes in B cells during pregnancy. (A) Volcano plots of DEGs in B cells (naïve B cells, memory B cells and plasmablast). Genes with |log2(FC)| ≥ 0.3, adjusted *p* < 0.05, related B‐cell activation and RNA splicing were labelled by gene symbols. (B) GO term enrichment of genes which highly expressed in different trimester compared to non‐pregnancy in B cell. Red means up‐regulation compared to non‐pregnancy, blue means down‐regulation compared to non‐pregnancy. (C) Dot plot of *BLNK*, *MS4A1* and *CD79B* in naïve B cells and memory B cells. Dots sizes represent the proportion of cells expressed in four stages. Dot colours represent average expression levels of monocytes in four stages. (D) Box plots of the cell scores of *BLNK*, *MS4A1* and *CD79B* in naïve B cells and memory B cells. Wilcoxon rank‐sum test was applied. (E and F) Box plots of the cell scores of two GO biological process terms (B‐cell receptor signalling pathway and B‐cell activation) in naïve/memory B cells and plasmablast across four conditions. (G and H) Box plots of the cell scores of two GO biological process terms (RNA splicing and alternative mRNA splicing via spliceosome) in naïve/memory B cells and plasmablast across four conditions. (I) *HNRNPL* expression pattern across four conditions; the graph on the right shows the significance of three periods of pregnancy compared to non‐pregnant controls. Wilcoxon rank‐sum test was applied. All differences with *p* < 0.05 are indicated. * *p* < 0.05, ** *p* < 0.01, *** *p* < 0.001, **** *p* < 0.0001, ns = not significant

Pregnancy is also associated with changes in B‐cell subsets. Compared with non‐pregnancy, the downregulated DEGs in the peripheral blood of pregnant women were mostly associated with B‐cell function (Figure 5A), with enrichment in the “B‐cell receptor signalling pathway” and “B‐cell activation” (Figure 5B). Moreover, many genes related to “signal transduction pathways”, “B‐cell activation” and “immune responses” were markedly downregulated throughout gestation (Figure S4A–E), including *MS4A1*, *CD79B* and *BLNK* (Figure 5C and 5D). The protein encoded by *MS4A1* is a membrane protein specific to B lymphocytes and plays a critical role in regulating the influx of cellular calcium necessary for B lymphocyte activation.^39^ *CD79B* cooperates with *CD79A* to initiate the signal transduction cascade activated by the B‐cell antigen receptor complex. *BLNK* encodes a cytoplasmic junction or adaptor protein important in B‐cell development. This protein is located downstream of the B‐cell receptor, which connects SYK kinase with various signalling pathways and regulates B‐cell function and development.^40^ To verify our results, we assessed the expression levels of two significant GO pathways (i.e., B‐cell receptor signalling pathway and B‐cell activation) in B cells (memory, naïve, plasmablast cells). Consistent with the above results, the pathway scores decreased significantly during pregnancy compared to the control group (Figure 5E and 5F). Compared to non‐pregnant women, most upregulated DEGs in the B cells were involved in RNA splicing‐related pathways during early gestation, although the number of genes related to RNA splicing decreased as pregnancy progressed (Figure 5A and 5B, S4H). The RNA splicing and alternative mRNA splicing via spliceosome pathway scores were also significantly elevated in B cells and plasmablasts during early‐mid gestation (Figure 5G and 5H), as was *HNRNPL* expression in the memory B cells (Figure 5I). These results suggest that upregulation of alternative splicing may contribute to the inhibition of B‐cell activity. In addition, to gain insight into the changes in B‐cell responses to IFN during pregnancy, we compared the score of ISGs (based on all collected ISGs) and response to type I IFN pathway across all four stages (Figure S4F and G). Results showed no significant change in B‐cell response to IFN during the first and second trimesters, but a slight increase in B‐cell response to IFN in the third trimester.

### 2.6 Global comparison analysis of communication among immune cells

**FIGURE 6:**
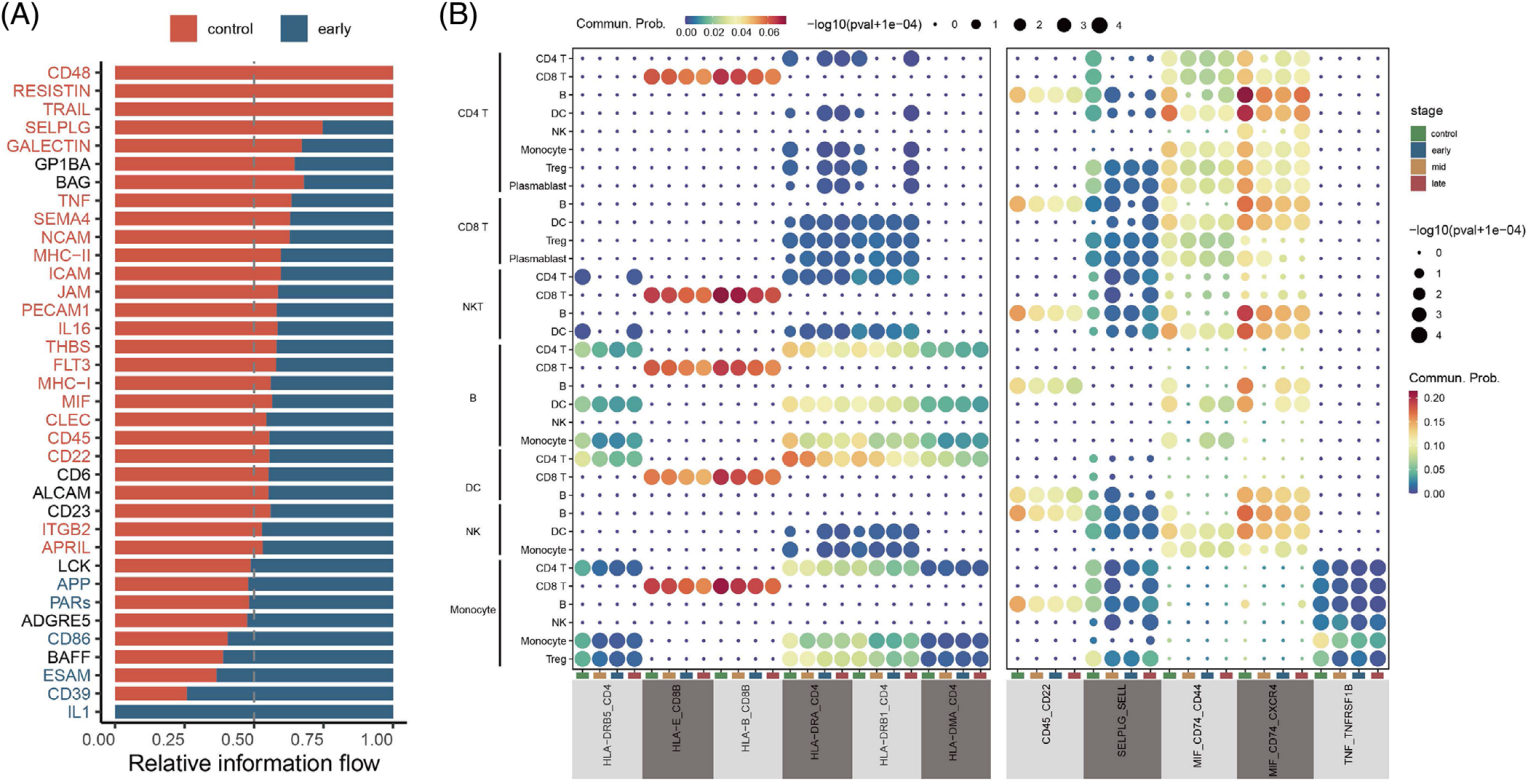
The interactions of peripheral immune cells in pregnant women. (A) Bar graph shows significant signalling pathways were ranked based on differences in the overall information flow within the inferred networks between first trimester and non‐pregnancy. The top signalling pathways coloured red are enriched in first trimester, and those coloured light blue were enriched in the non‐pregnancy. (B) Dot plot of the predicted interactions between immune cell types in the first trimester and in non‐pregnant control. *p* values are indicated by the circle sizes, as shown in the scale (permutation test). The means of the average expression level of interacting molecule 1 in cluster 1 and interacting molecule 2 in cluster 2 are indicated by the colour

Complex cellular responses are triggered by ligand‐receptor binding and the subsequent activation of specific signalling pathways. To identify differences in molecular interactions between the major immune cell types in pregnant versus non‐pregnant women, we conducted bioinformatics analysis of cell‐cell communication using CellChat.^41^ Results showed that the number of inferred interactions and interaction strength decreased obviously during gestation (Figure S5A). The overall signalling patterns determined by CellChat showed that signalling and immune activity‐related pathways, such as the SELPLG, GALECTIN, MHC‐II, MHC‐I, IL16, CD45, TNF, LCK and MIF signalling pathways (Figure 6A), were significantly downregulated, in agreement with our previous results. Furthermore, we identified 11 specific ligand‐receptor pairs involved in immune cell communication that were significantly decreased in pregnancy compared to healthy controls (Figure 6B). Specifically, many ligand/receptor pairs associated with T‐cell signalling were downregulated during gestation, including HLA‐DRB1/CD4, HLA‐DRA/CD4, HLA‐DRB5/CD4, HLA‐DMA/CD4, HLA‐E/CD8B and HLA‐B/CD8B. Notably, the downregulation of CD45/CD22 was highly significant in the B‐B, CD4T‐B, CD8T‐B, NKT‐B, DC‐B, NKT‐B and monocyte–B‐cell interactions throughout pregnancy (Figure 6B). CD22 is an inhibitory co‐receptor on the B‐cell surface that inhibits B‐cell receptor‐induced signalling,^42^ which can be reversed by CD45 to maintain tonic B‐cell antigen receptor signalling.^43^ These results demonstrate that B‐cell signalling is attenuated during pregnancy, in part due to weakened CD45/CD22 interactions. Furthermore, CD45 and CD22 contribute to a broad spectrum of immune cell interactions, through which B cells interact with CD4 T, CD8 T, NKT, DC, NK and monocyte cells. In addition, monocytes were the prominent influencer controlling TNF signalling (primarily TNF‐TNFRSF1B) (Figure 6B, S5B), with TNF/TNFRSF1B markedly decreased during pregnancy (Figure 6B). This may contribute to monocyte dysfunction during pregnancy.

The SELPLG and MIF signalling pathways play important roles in immune cell activation and migration.^44^, ^45^ Here, CellChat predicted that the SELPLG and MIF signalling pathways were downregulated during pregnancy (Figure S5C and D). Moreover, interactions of ligand/receptor pairs involved in these pathways, such as SELPLG/SELL, CD74/CXCR4 and CD74/CD44, also decreased significantly (Figure 6B). The signalling pathways corresponding to ligand/receptor pairs covered almost all immune cells (Figure 6B). This cell–cell communication analysis highlighted that cell signalling pathways and immune cell interactions are attenuated across pregnancy, and interactions between immune cells are complex and redundant.

### 2.7 Transcriptomic clock of normal pregnancy identified by machine learning

**FIGURE 7:**
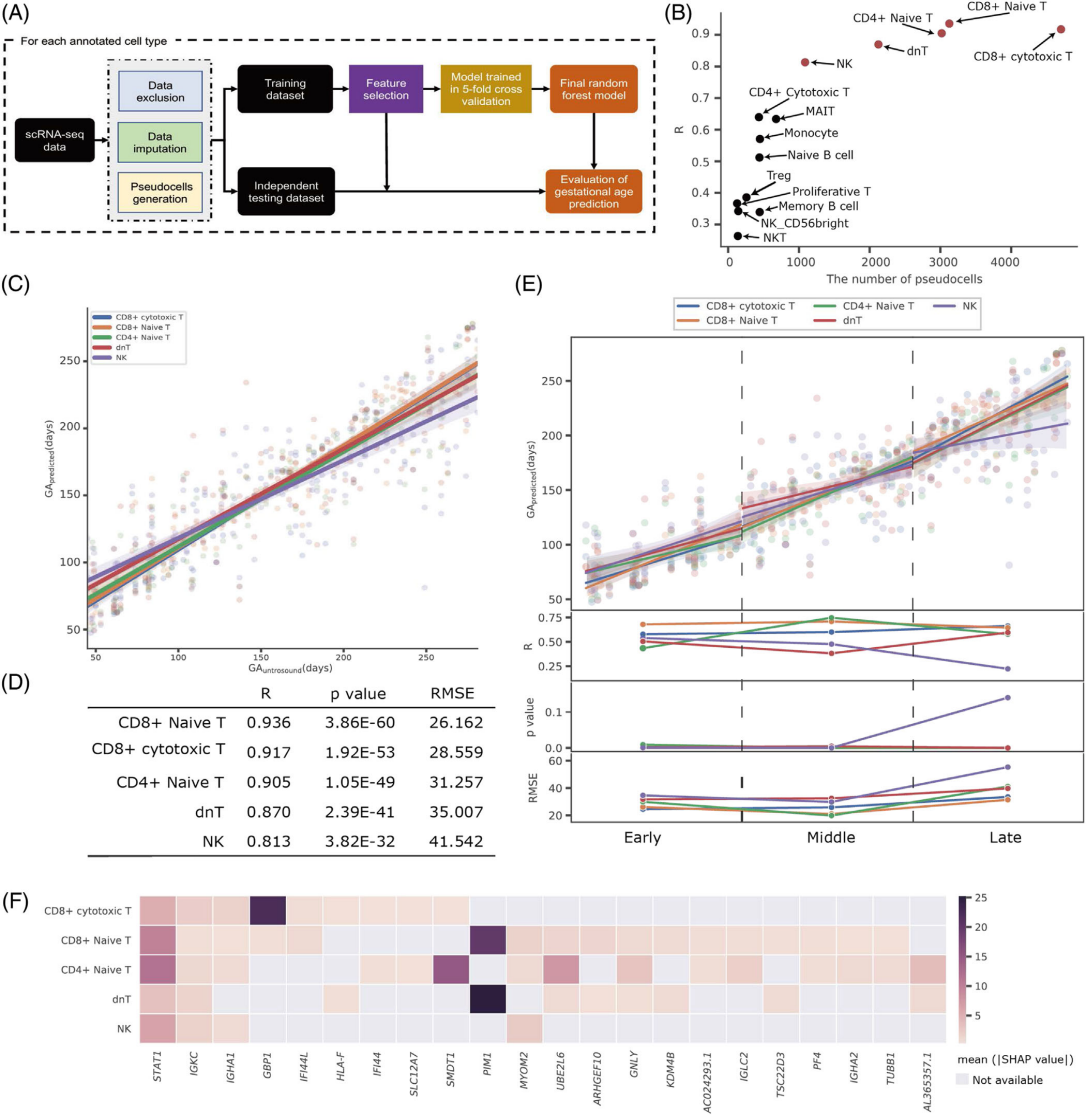
Machine learning models to establish transcriptomic clock of normal pregnancy. (A) Flowchart of developing the cell‐type‐specific machine learning models. (B) Pearson's correlation coefficient (*R*) values demonstrating the correlation of the predicted gestational age in days ($GA_{predicted}$) by the 14 cell‐type‐specific models based upon the independent testing datasets with the estimates by the first‐trimester ultrasound ($GA_{ultrasound}$). The *x*‐axis represented the number of pseudo‐cells. The red circle points indicated the five models with the *R* values greater than 0.8. (C and D) The correlation of the $GA_{predicted}\;$ by the five predominant cell‐type‐specific models with the $GA_{ultrasound}\;$ during the entire pregnancy period (C), as well as the performance metrics in terms of *R* values, *p* values and root mean squared errors (RMSE) (D). (E) The correlation of the $GA_{predicted}\;$ with the $GA_{ultrasound}$ (plot in the first row), *R* values (plot in the second row), *p* values (plot in the third row) and RMSE values (plot in the last row) during the periods of early, middle and late pregnancy. (F) Top 22 genes involved in at least two of the five predominant cell‐type‐specific models were ranked and were prioritised by the mean absolute of SHAP values. The first two genes of *STAT1* and *IGKC* were involved in all five predominant cell‐type‐specific models

Next, we investigated the utilization of scRNA‐seq data to establish a transcriptomic clock and thereby predict gestational age of normal pregnancy (Figure 7A). After exclusion and imputation, 14 out of 18 cell types from the 131 subjects were obtained, with the number of single cells ranging from 1,268 to 47,043 (Table S3A). For each cell‐type‐specific subset, we generated pseudo‐cells and conducted data splitting, leading to 14 pairs of training datasets and independent testing datasets containing 131–3, 234 and 126–1, 351 pseudo‐cells, respectively (Table S3A). By applying the LASSO (least absolute shrinkage and selection operator) algorithm, 147–1, 615 gestational age‐relevant genes were selected (Table S3A). We trained 14 random forest (RF)‐based regression models to predict gestational age (in days) using the corresponding 14 cell‐type‐specific training datasets, with the optimal hyperparameters determined in a fivefold cross validation (Table S3B). We then applied the final‐trained cell‐type‐specific models to the independent testing datasets to yield predicted gestational age ($GA_{predicted}$) estimates, which were compared with the estimates obtained by first‐trimester ultrasound ($GA_{ultrasound}$) using Pearson's correlation coefficients (*R*). We filtered out models with *R* values less than 0.8 (Figure 7B), leading to the top five prediction models built upon the CD8+ naïve T (*R* = 0.936, *p* = $3.86\;\times \;10^{-60}$, RMSE = 26.162), CD8+ cytotoxic T (*R* = 0.912, *p* = $1.05\;\times \;10^{-51}$, RMSE = 29.314), CD4+ naïve T (*R* = 0.905, *p* = $1.05\;\times \;10^{-494}$, RMSE = 31.257), dnT (*R* = 0.867, *p* = $8.43\times \;10^{-41}$, RMSE = 35.363) and NK cell types (*R* = 0.813, *p* = $3.82\;\times \;10^{-32}$, RMSE = 41.542) (Figure 7C and 7D). We further assessed the prediction capabilities of the models in early, middle and late pregnancy, respectively. Results showed that the top five cell‐type‐specific models in gestational age prediction were more accurate in middle pregnancy than in early or late pregnancy (Figure 7E). In particular, compared to the other cell types, CD4+ and CD8+ naïve T cells in middle pregnancy showed the highest prediction ability, with RMSE values of 19.823 and 21.030, respectively. These results suggested that CD8+ naïve T, CD8+ cytotoxic T, CD4+ naïve T, dnT and NK cell types in PBMCs during normal pregnancy, especially in the second trimester, have the potential to predict gestational age with high accuracy.

We then examined feature importance of the five cell‐type‐specific models by calculating the mean absolute values of SHAP (SHapley Addtive exPlanations).^46^ A total of 22 genes involved in at least two of the five cell‐type‐specific models were then ranked and prioritized. Among these genes, *STAT1* and *IGKC* exhibited high importance across all five cell types when predicting gestational age (Figure 7F). In addition, we performed GO enrichment analysis of the 159 genes involved in any of the five cell‐type‐specific models. Results indicated that these genes were significantly enriched in immune terms, including “T cell activation”, “immune response‐activating signal transduction”, “B cell activation” and “cellular response to interferon‐gamma” (Figure S6). These findings were in line with our above analyses.

## 3 DISCUSSION

Pregnancy is accompanied by significant systemic immunological adaptations.^9^ However, previous studies have primarily focused on classical experimental methods such as blood cell count and flow cytometry, making it difficult to obtain comprehensive scenarios of cellular and molecular immune responses during gestation. To address this issue, we profiled the immune landscape in PBMCs from 6–40 weeks of gestation at single‐cell resolution and determined the dynamic nature of cellular responses in human pregnancy.

During pregnancy, the maternal immune system is altered to protect allogeneic fetal tissues against premature rejection. Monocytes increase during pregnancy, beginning in the first trimester,^14^ and exhibit anti‐inflammatory activity.^18^ For example, Susanne et al. reported that monocytes are in an inhibitory state as LPS‐induced IL‐12 and TNFα production by monocytes is decreased compared to that in non‐pregnancy.^47^ In contrast, we observed that many genes related to the “cell chemokine” and “positive regulation of cell activation” pathways, such as *CD83*,^48^ were markedly downregulated in the three periods of pregnancy, although IFN responses were activated at the early stage (GW6–9). Cell communication analysis showed the pair TNF/TNFRSF1B from monocyte to most immune cells (especially monocytes) markedly decreased during pregnancy, which may contribute to immune cell dysfunction during pregnancy. Additionally, CD83 can upregulate PGE2 expression in monocytes, which can, in turn, suppress T‐cell immune responses.^26^ Thus, we propose that monocytes play an essential role in maintaining maternal‐fetal immune balance.

Although T‐cell response is generally believed to be suppressed during pregnancy based on the symptoms alleviated in some patients with autoimmune diseases, the mechanism is yet unclear.^9^, ^49^ Here, we verified the suppression of T cells and explored the underlying mechanism. Notably, T‐cell activity was significantly dampened throughout pregnancy (although IFN responses were enhanced), possibly due to the significant decrease in the expression of TCR‐CD3 complexes (*TRAC*, *TRBC1*, *TRBC2*, *CD3D*, *CD3E* and *CD3G*) and ligation of co‐stimulatory molecules (*CD2* and *CD27*), essential components for effective T‐cell activation,^30^ in CD4+ and CD8+ T cells. Our results also revealed that many cytotoxic‐related genes were downregulated, whereas exhaustion‐related genes, such as *LAG3*, were upregulated in CD4+ and CD8+ T cells throughout pregnancy, which further illustrated T‐cell functionality impairs. LAG3 has a negative regulatory effect on T cells and in combination with PD1 can mediate a state of exhaustion.^50^ Our results also showed that circulating B cells were greatly reduced during the third trimester, probably due to the elevated level of estrogen.^51^ There was a trend toward reduction in naïve B cells in our data, although this was not statistically significant. B‐cell function is known to decrease during pregnancy,^9^ in agreement with our results. Cell‐cell communication analysis also showed attenuation of CD45/CD22 interactions, which play an important role in B‐cell antigen receptor signalling,^43^ thus contributing to maternal‐foetal immune tolerance.

The RNA splicing and alternative mRNA splicing via spliceosome pathway scores were elevated significantly in B and T cells in the first trimester, but gradually decreased as pregnancy progressed. Alternative splicing plays an important role in maintaining T‐ and B‐cell homeostasis in the peripheral immune system.^36^, ^37^ For example, PTPRC, one of the genes identified in T and B cells to undergo alternative splicing,^52^ plays a crucial role in T‐ and B‐cell activation and subsequent proliferation and cytokine production.^53^, ^54^, ^55^ Moreover, *HNRNPL*, an encoded RNA‐binding protein that regulates alternative splicing of PTPRC, resulting in different CD45 protein isoforms and functions,^37^ was also upregulated in T and B cells during pregnancy. These results suggest that alternative mRNA splicing may play an important role in regulating T‐ and B‐cell activity during pregnancy. However, the specific mechanism remains unclear. In addition, scRNA‐seq data cannot be used to identify protein isoforms, and protein sequencing is required to learn more about how alternative splicing regulates T‐cell activity during pregnancy.

Both NK and T cells play critical roles in maintaining maternal‐fetal balance.^56^, ^57^ In addition, T and NK cell responses are vital for the control and clearance of viruses.^58^, ^59^ The functions of NK and T cells were thought to be inhibited during pregnancy to protect the fetus,^49^, ^60^ but recent studies have proposed an alternative view. Notably, several studies have demonstrated enhanced NK and CD4+ T‐cell responsiveness to type I IFN^8^ and pH1N1 virus during pregnancy.^61^ Similarly, our results showed that IFN/virus responses in NK (NK/NK_CD56‐bright) and T cells (CD4+ cytotoxic T, CD4+ naïve T, CD8+ cytotoxic T, CD8+ naïve T, NKT, Treg cells) increased significantly and progressively during pregnancy, reaching a peak in the third trimester (especially NK cells). Additionally, our results indicate *STAT1* may play an important role in activating immune response to interferon in NK/NK_CD56 bright cells during pregnancy. An excessive response to IFN/virus during pregnancy may induce a reversal in immune response from a healthy to destructive status, leading to increased disease severity. Consistent with this idea, numerous chemokines are reported to be related to increased pathogenicity and morbidity in influenza infection in humans.^62^ Furthermore, blocking influenza‐induced cytokines can prevent influenza death in mice without increasing virus titres in infected tissues.^63^ These results indicate that the maternal immune system induces fine immune regulation for maintenance of pregnancy.

We also developed cell‐type‐specific models to predict gestational age (in days) of normal pregnancy. Five cell types (i.e., CD8+ cytotoxic T, CD8+ naïve T, CD4+ naïve T, dnT and NK cells) exhibited high accuracy in gestational age prediction. Other cell types showed relatively low prediction ability, which may be due to the small number of captured single cells. Thus, further analysis should be conducted using these cell types with sufficient data. Furthermore, the predictive models showed higher accuracy in middle pregnancy than in early or late pregnancy, suggesting a steady state of cell evolution during the middle pregnancy period.

This study has several limitations. First, our study lacked postpartum data, and the identified alterations in immune response during pregnancy need to be further validated using such samples. Second, different gestational weeks corresponded to different participants in our study, thus increasing cohort heterogeneity. However, we were able to capture the dynamic nature of cellular responses during gestation in our data, further illustrating the universality of maternal PBMC immune adaptations. Future work should extend and validate our results by examining maternal PBMC immunological changes in the same woman from pre‐pregnancy to postpartum.

To the best of our knowledge, this is the first study to visualise the dynamic landscape of maternal PBMC immune adaptations throughout pregnancy at the single‐cell resolution. This work should help improve our understanding of the pathophysiological reactions during pregnancy and lay a foundation for linking unfavourable outcomes of mother and child to the maternal immune system during gestation.

## 4 METHODS

### 4.1 Ethics statement

All sample collection and research protocols were performed with the approval of the Institutional Review Board on Ethics Committee of BGI (approval reference number BGI‐IRB 21082). The PBMCs were collected after obtaining written informed consent from donor patients. All procedures followed the ‘Interim Measures for the Administration of Human Genetic Resources’.

### 4.2 Inclusion and exclusion criteria

Inclusion criteria: Primiparous women aged 20–34 years, diagnosed with singleton pregnancy, first pregnancy, no abortion, no drug abortion or ectopic pregnancy. Pregnant women registered in Shenzhen Maternity and Child Healthcare Hospital at 6–8 weeks of gestation. No pregnancy complications.

Exclusion criteria: Various types of chronic diseases, especially immune system diseases, including but not limited to tumour, asthma, rheumatism, lupus erythematosus, history of hyperthyroidism and history of hypothyroidism. Appearance during pregnancy: subclinical hypothyroidism during pregnancy, ICP, GDM, HDP, gestational thrombocytopenia, FGR and macrosomia in middle and late stages of pregnancy (ICP: intrahepatic cholestasis of pregnancy, GDM: gestational diabetes mellitus, HDP: hypertensive disorders of pregnancy, FGR: fetal growth restriction).

### 4.3 PBMC collection and treatment

Peripheral blood samples (3 ml) were collected and gently rotated to mix thoroughly. Whole blood was first diluted by adding an equal amount of sterile phosphate‐buffered saline (PBS) (Cat. No. 10010–031) to a 15‐ml conical centrifuge tube, added 3 ml of Histopaque‐1077 (Cat. No. 10771, 6 × 100 ml) and brought to room temperature. The diluted whole blood was layered onto Histopaque‐1077, then horizontally centrifuged at 500 *g* for 20 min at 20°C. The middle mononuclear cell layer was transferred to a new 15‐ml centrifuge tube, with 5 ml of 1% bovine serum albumin (BSA) and PBS then added for washing. The solution was centrifuged at 300 *g* for 10 min and the supernatant was discarded. After this, 10 ml of 1% BSA (with PBS) was added to wash the cells, followed by centrifugation at 300 *g* for 5 min and the addition of 2 ml of 1% BSA (PBS) to resuspend the cells. Trypan blue was used (0.4%) (Cat. No. C0040, 100 ml) (cell solution: trypan blue = 1:1) to calculate and count cell viability and number under a microscope, with cell viability shown to be greater than 90%. Approximately, 100,000 cells were centrifuged at 300 *g* for 5 min, with 100 μl of Cell Resuspension Buffer (Cat. No. 1000019895) then added to resuspend the cells.

### 4.4 scRNA‐seq library preparation and sequencing

ScRNA sequencing libraries were constructed using DNelabC4 following the manufacturer's instructions.^64^ The libraries were quantified using a Qubit ssDNA analysis kit (Thermo Fisher Scientific) and sequenced using the DIPSEQ T1 sequencer of the China National Gene Bank (CNGB).

### 4.5 Processing of raw scRNA‐seq data

Raw sequencing data were processed by PISA. Seurat v4.0.1 was applied for downstream analysis. We filtered the data using the following criteria: (1) Cells with gene expression <800 or >6000 were discarded and (2) Cells with a mitochondrial gene percentage >5% were filtered out.

### 4.6 Multiple dataset integration

We adopted the package integration method described in https://satijalab.org/seurat/articles/integration_introduction.html. Seurat v.4.0.4 was used to combine different scRNA‐seq datasets into an integrated and unbatched dataset. In the first step, we identified 2000 features with high cell‐to‐cell variability. In the second step, we used the FindIntegrationAnchors function to identify ‘anchors’ between a dataset, and input these anchors into the IntegrateData function to create an aggregated matrix of all cells.

### 4.7 Cluster‐specific gene identification and GO enrichment analysis

The FindAllMarkers function in Seurat was used to identify cluster‐specific marker genes (thresh.use = 0.25, min.pct = 0.25, only.pos = TRUE). The R package clusterProfiler^65^ was employed for GO term enrichment of cluster‐specific genes and the BH method was used for multiple test correction. Both GO and Kyoto Encyclopedia of Genes and Genomes (KEGG) terms with *p* < 0.05 were considered significantly enriched.

### 4.8 Defining cell state score

We use cell scores to assess how well a single cell expresses a certain set of pre‐defined expressed genes.^66^ Cell scores were initially based on the average expression of the predefined gene set in each group of cells. The AddModuleScore function in Seurat was used to implement the method with default settings. We used response to type I interferon (GO: 0034340), defense response to virus (GO: 0051607), T‐cell receptor signalling pathway (GO: 0050852), T‐cell activation (GO: 0042110), B‐cell receptor signalling pathway (GO: 0050853), B‐cell activation (GO: 0042113), RNA splicing (GO:0008380), alternative mRNA splicing via spliceosome (GO:0000380), 58 ISGs,^67^ 11 cytotoxicity‐associated genes (*PRF1*, *IFNG*, *NKG7*, *GZMB*, *GZMA*, *GZMH*, *KLRK1*, *KLRDC1, CTSW* and *CST7*,), 13 apoptosis associated genes (*TNFSF10*, *TRADD*, *FAS*, *FASLG*, *FADD*, *TNFSF14*, *BAD*, *BAX*, *CASP4*, *DAP3*, *DAXX*, *PDCD10* and *PDCD6*) and 5 well‐defined exhaustion markers (*LAG3*, *PDCD1*, *CTLA4*, *HAVCR2* and *TOX*) to define the response to type I interferon, defense response to virus, T‐cell activation, B‐cell activation, RNA splicing, alternative mRNA splicing via spliceosome, ISG, cytotoxicity and exhaustion score, respectively.

### 4.9 Cell communication

The R packet CellChat^41^ was used for cell communication analysis with default parameters. Cell ChatDB.human was used for our datasets.

### 4.10 Machine learning model development

#### 4.10.1 Data pre‐processing and splitting

We excluded annotated cell types with fewer than 1,000 cells and imputed zero expression genes using the MAGIC algorithm v3.0.0.^68^ Expression data were normalized by removing the mean from each gene expression and scaling to unit variance. Women with multiple single‐cell sequencing data at different time during the pregnancy period were treated as different subjects with distinct gestational ages. To build the machine learning models to predict gestational age at the cell‐type level, a subset of scRNA‐seq data for each cell type was constructed. For each cell‐type‐specific subset, we created a training dataset and an independent testing dataset using the stratified random splitting approach in a 7:3 ratio. The stratified sampling method was used to ensure similar scRNA‐seq data distribution per subject in both training and testing datasets. As subjects could possess many single cells, it is undesirable to build a predictive model based on such a large number of single cells of the same gestational age. Hence, we computed median gene expression for every 10 single cells randomly sampled (without replacement) from the same subject, denoted as pseudo‐cells.

#### 4.10.2 Machine learning model training and evaluation

In the training dataset, the LASSO algorithm (scikit‐learn v0.24.2: Lasso), with the penalty parameter value of 0.1 set empirically, was employed to select gestational age‐relevant genes. Subsequently, a random forest (RF) regression model (scikit‐learn v0.24.2: RandomForestRegressor) was trained and optimised by tuning the hyperparameters to minimise root mean squared error (RMSE) using a random search strategy with fivefold cross validation (scikit‐learn v0.24.2: RandomizedSearchCV). After determining the optimal hyperparameters of the RF models, we re‐trained the final RF regression model to predict gestational age using the entire training set.

The independent testing dataset was used to evaluate performance of the predictive model. We obtained the predicted gestational age of a subject by averaging the outputs of the final RF regression model based on the pseudo‐cells of the subject. Pearson's correlation coefficients (*R*) and RMSE were used as the evaluation metrics.

We used the SHAP (SHapley Addtive exPlanations, v0.39.0) ^46^ to determine the importance of genes in prediction of gestational age. Each SHAP value measured the change in the predicted value of the gestational age of subject *i* attributed to gene *j*. Mean absolute SHAP values across all subjects in the dataset represented the overall importance of a particular gene in the prediction of gestational age by the final RF regression model. Larger mean absolute SHAP values of a gene represented higher contributions to gestational age prediction. Genes were initially ranked by the mean absolute SHAP values calculated from the training dataset. The optimal number of genes was then determined by the minimum MSE. We then retrained the models using the optimal hyperparameters and genes as the final models.

## CONFLICT OF INTEREST

The authors declare no conflict of interest.

## Supporting information

Supporting Information
