# Supplementary material for: Single‐cell atlas of peripheral blood mononuclear cells from pregnant women
Source: Clin Transl Med. 2022 May 6;12(5):e821. doi: 10.1002/ctm2.821 (PMC9076016; doi:10.1002/ctm2.821)
Supplement: Supplementary file 1 — Supporting Information [file CTM2-12-e821-s001.pdf]

Supplementary Materials for

Single-cell atlas of peripheral blood mononuclear cells from pregnant women

Dongsheng Chen†, Wei Wang†, Linlin Wu†, Langchao Liang†, Shiyou Wang†, Yunfeng Cheng†, Tongda Zhang, Chaochao Chai, Qiuhong Luo, Chengcheng Sun, Wandong Zhao, Zhiyuan Lv, Ya Gao, Xiaoxia Wu, Ning Sun, Yiwei Zhang, Jing Zhang, Yixuan Chen, Jianing Tong, Xiangdong Wang*, Yong Bai*, Chaoyang Sun*, Xin Jin*, Jianmin Niu*

This file includes:

Figure S1. Single cell data display for each library and GO term for all cell types (related to Figure 1)
Figure S2. Transcriptomic profiling of NK cells and monocytes during pregnancy (related to Figure 3)
Figure S3. Transcriptomic profiling of T cells throughout gestation (related to Figure 4)
Figure S4. Changes in B cell activity during pregnancy (related to Figure 5)
Figure S5. PBMC cell communication in pregnancy and non-pregnancy (related to Figure 6)
Figure S6. Enriched GO terms for genes involved in the five cell-type-specific models toward gestational age prediction (related to Figure 7)
Table S1. Basic information of the enrolled subjects (related to Figure 1)
Table S2. Cell composition of each sample (related to Figure 2)
Table S3. Machine learning model (related to Figure 7)

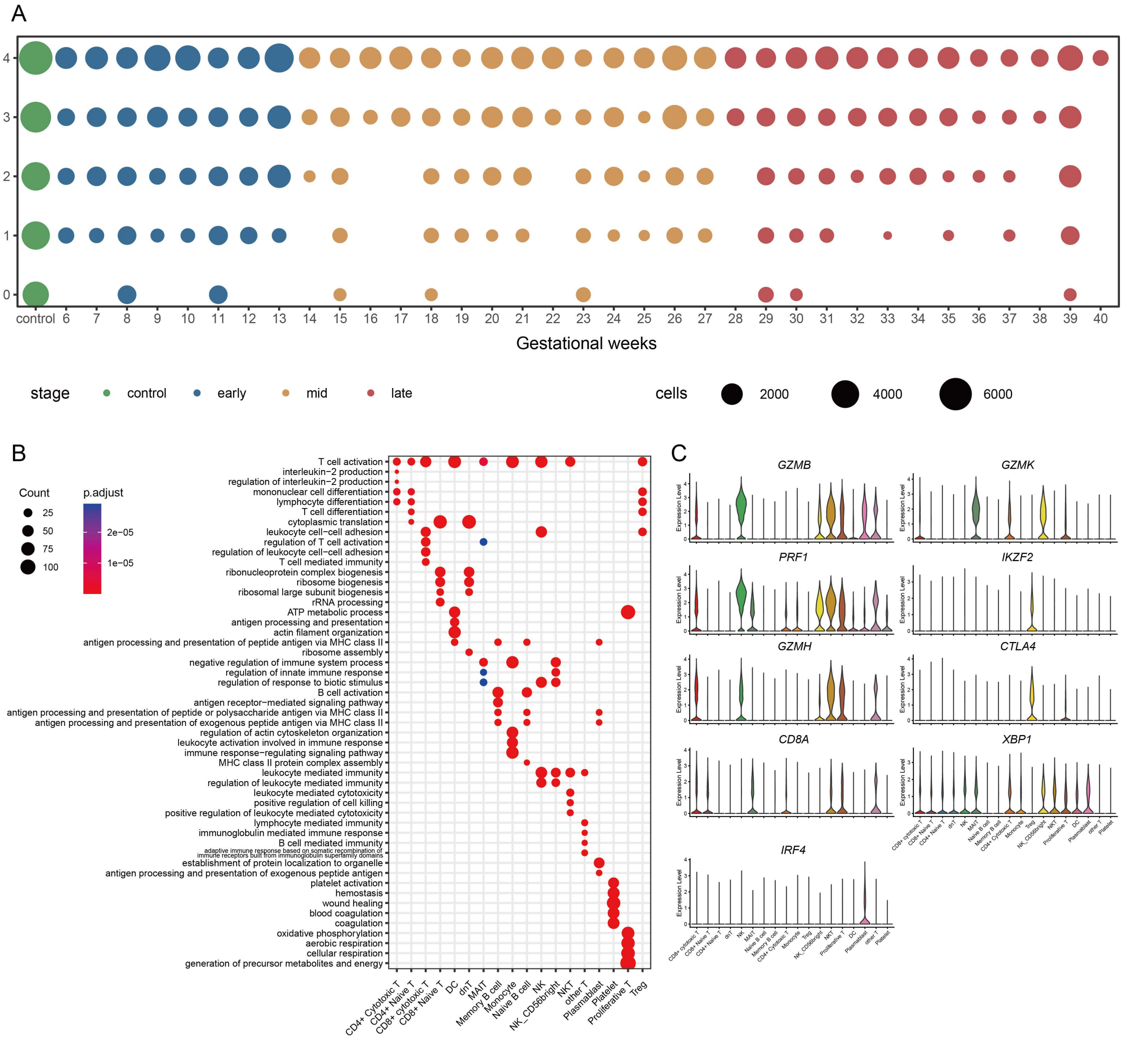

FIGURE S1 Single cell data display for each library and GO term for all cell types (related to Figure 1). (A) Schematic diagram of sample collection. The abscissa axis indicates gestational weeks (GW). Control means subjects who are not pregnant (n=5). Each point represents a subject. The size of the point indicates the number of PBMC collected by a subject, and the color indicates the pregnancy period of the subject. early= the first trimester (GW6-13) (n=34), mid= the second trimester (GW14-27) (n=52), late= the third trimester (GW28-40) (n=45). (B) GO term enrichment for differentially expressed genes in all cell types. The p adjusted values lower, point redder. Point size represents the number of DEGs involved in specific GO terms. (C) Violin plots of expression values for other cell type-specific marker genes.

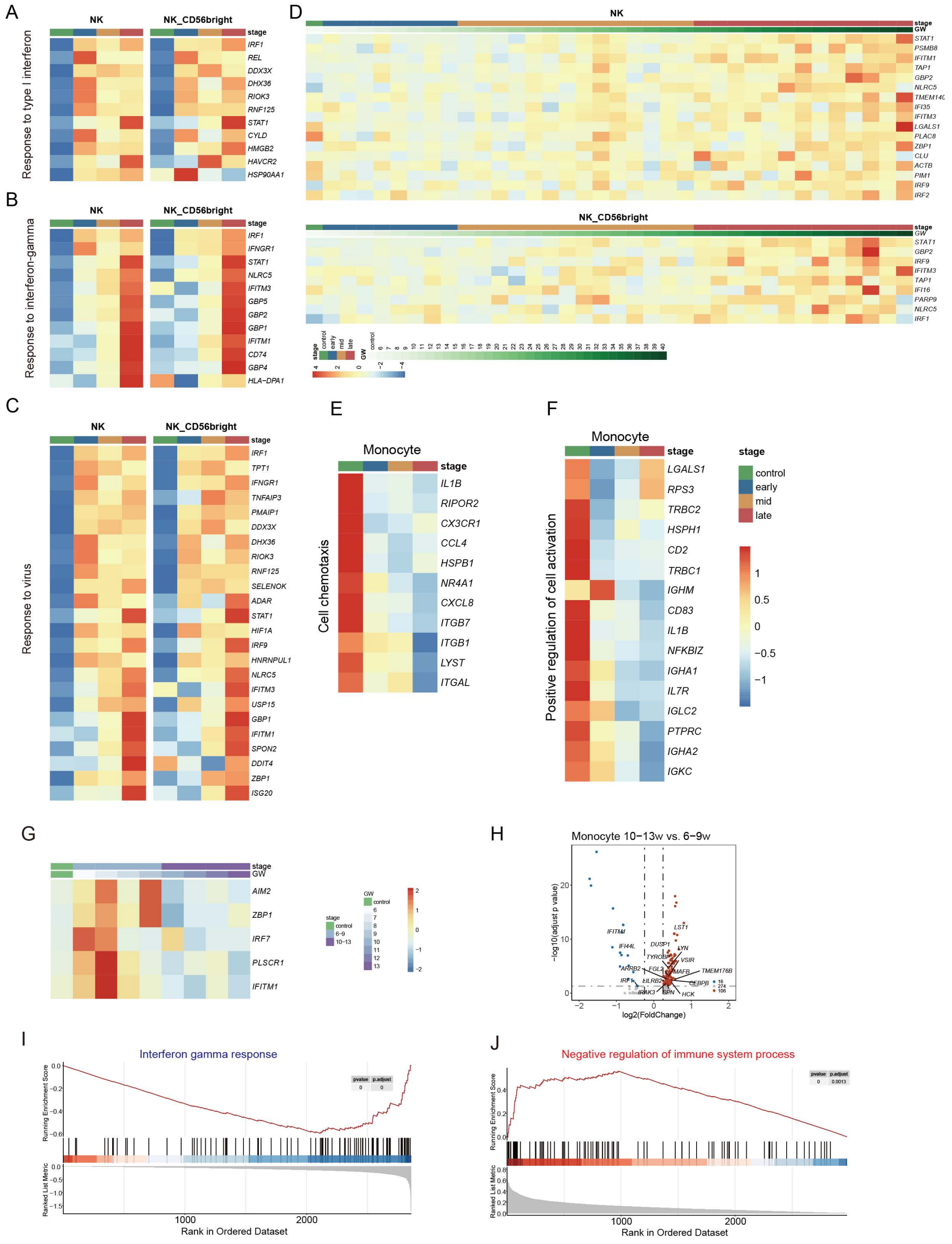

FIGURE S2 Transcriptomic profiling of NK cells and monocytes during pregnancy (related to Figure 3). (A) Heatmap of DEGs enriched in "response to type I interferon", (B) "response to interferon-gamma", (C) "response to virus" in NK/NK_CD56bright cells across four conditions. Colors represent scaled genes expression level, and higher expression is red and lower expression is blue. (D) Heatmap of ISGs across four stages in NK/NK_CD56bright cells. Colors represent scaled genes expression level, and higher expression is red and lower expression is blue.

(E-F) Heatmap of DEGs enriched in "cell chemotaxis", "positive regulation of cell activation" across four stages in monocytes. (G) Heatmap of the ISGs, including *AIM2*, *ZBP1*, *IRF7*, *PLSCR1* and *IFITM1* in monocytes derived from GW6-13 and pre-pregnant control samples. (H) Volcano

plots of DEGs of monocyte in GW6-9 and GW10-13. (I-J) GSEA results of "interferon gamma response" and "negative regulation of immune system process".

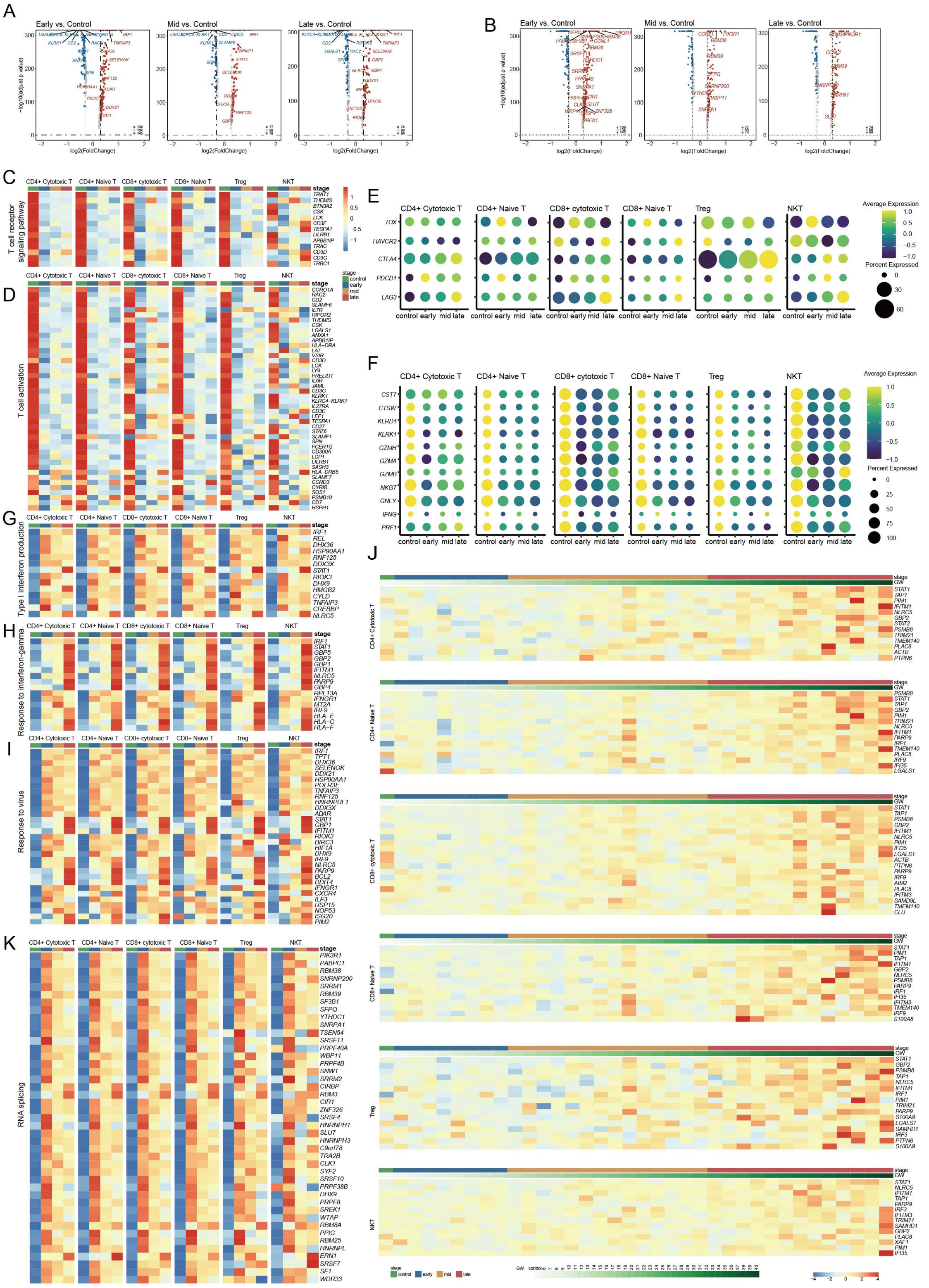

FIGURE S3 Transcriptomic profiling of T cells throughout gestation (related to Figure 4). (A) Volcano plots of differentially expressed genes in T cells. Interferon-related genes and T cell activation-related genes are labeled. (B) Volcano plots of differentially expressed genes in T cells. RNA splicing related genes are labeled. (C-D) Heatmap of DEGs enriched in "T cell receptor

signaling pathway”, “T cell activation” in T cells across four conditions. (E-F) Dot plot showing expression of genes associated with exhausted and cytotoxic in T cells across four conditions. (G-I) Heatmap of DEGs enriched in “type I interferon production”, “response to interferon-gamma”, “response to virus” in T cells across four conditions. (J) Heatmap of ISGs among every gestational week. Colors represent scaled genes expression level, and higher expression is red and lower expression is blue. (K) Heatmap of DEGs enriched in “RNA splicing” in T cells across four conditions.

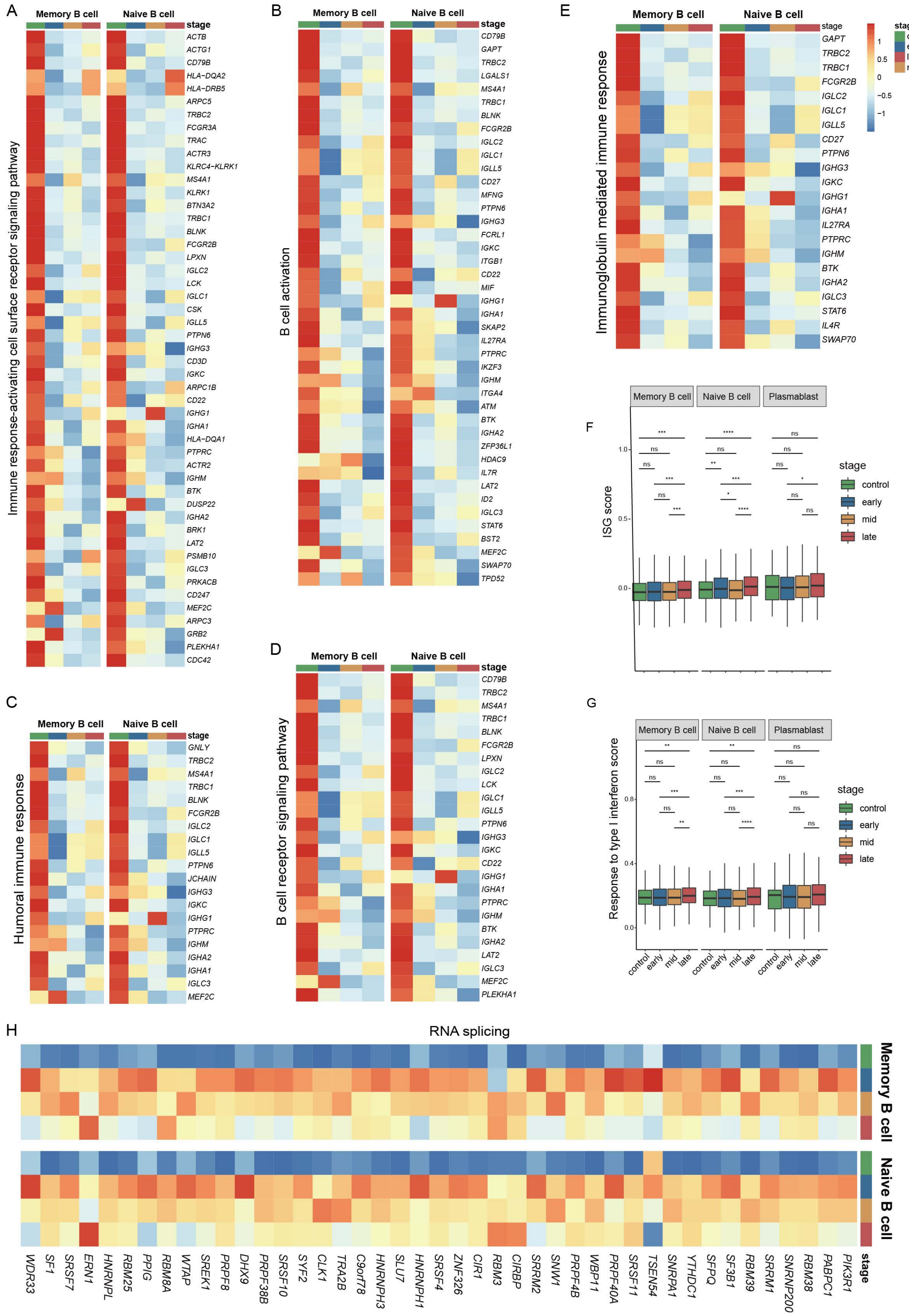

FIGURE S4 Changes in B cell activity during pregnancy (related to Figure 5). (A) Heatmap of DEGs enriched in “immune response-activating cell surface receptor signaling pathway”, (B) “B cell activation”, (C) “humoral immune response”, (D) “B cell receptor signaling pathway”, (E) “immunoglobulin mediated immune response” in Naïve/Memory B cells across four conditions. (F) Box plots of the collected ISGs scores across different clusters and conditions. Statistical significance of difference in pairs among four pregnant stages were labeled at the top of box plot.

Wilcoxon rank-sum test was applied. (G) Box plots of the cell scores of GO biological process terms (response to type I interferon) in B cells across four conditions. Wilcoxon rank-sum test was applied. All differences with $p < 0.05$ are indicated. *$p < 0.05$, **$p < 0.01$, ***$p < 0.001$, ****$p < 0.0001$, ns= not significant. (H) Heatmap of DEGs enriched in “RNA splicing” in Naïve/Memory B cells across four conditions.

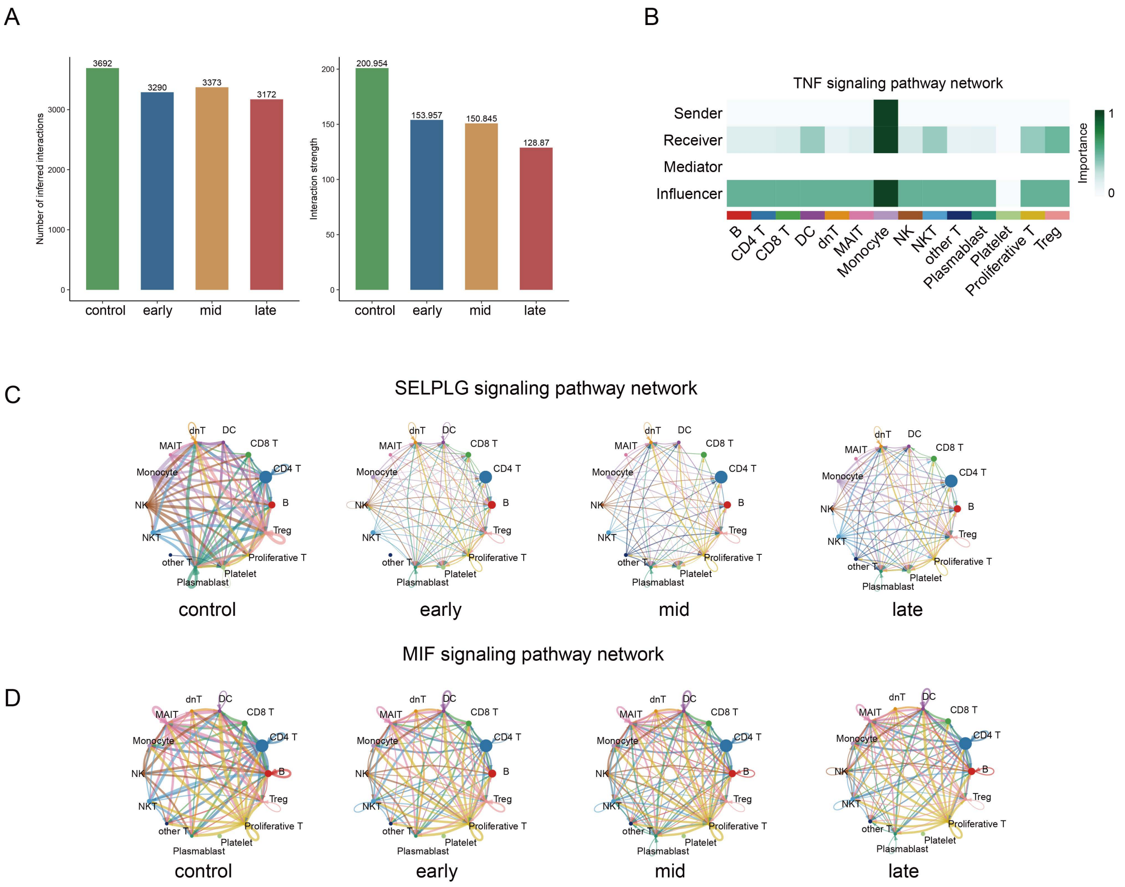

FIGURE S5 PBMC cell communication in pregnancy and non-pregnancy (related to Figure 6). (A) The bar chart shows that the total number of interactions of the inferred cell-cell communication networks from different conditions. (B) Heatmap shows centrality scores of TNF signaling pathway network. (C-D) SELPLG signaling pathway network and MIF signaling pathway network in different conditions.

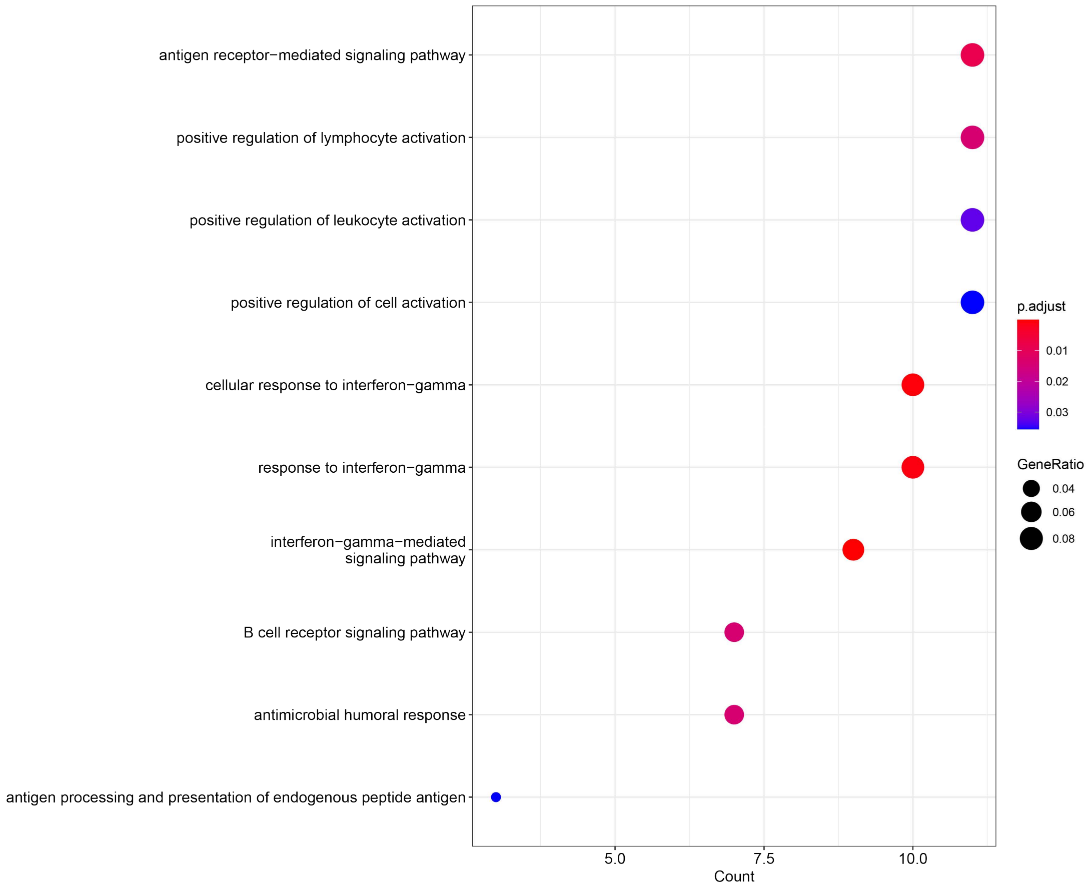

FIGURE S6 Enriched GO terms for genes involved in the five cell-type-specific models toward gestational age prediction (related to Figure 7)

Supplementary Table 1. Basic information of the enrolled subjects

	Control	Early	Mid	Late
	non-pregnancy	the first trimester (GW6-13)	the second trimester (GW14-27)	the third trimester (GW28-40)
	N=5	N=34	N=52	N=45
**Demographics**				
**Age，years**	25±1.9	29.7±2.7	28.7±2.6	28.7±3.2
**BMI, kg/m²**	22.85±3.05	20.65±2.01	22.51±1.95	25.62±2.47
**Previous births, No. (%)**				
Yes	0(0)	0(0)	0(0)	0(0)
No	5(100)	34(100)	52(100)	45(100)
**Abortion, drug abortion, and ectopic pregnancy, No. (%)**				
Yes	0(0)	0(0)	0(0)	0(0)
No	5(100)	34(100)	52(100)	45(100)
**Pregnancy complications, No. (%)**				
Yes	0(0)	0(0)	0(0)	0(0)
No	5(100)	34(100)	52(100)	45(100)
**Immune system diseases, No. (%)**				
Yes	0(0)	0(0)	0(0)	0(0)
No	5(100)	34(100)	52(100)	45(100)
**Appears during pregnancy, No. (%)**				
**Subclinical hypothyroidism**				
Yes	0(0)	0(0)	0(0)	0(0)
No	5(100)	34(100)	52(100)	45(100)
**ICP**				
Yes	0(0)	0(0)	0(0)	0(0)
No	5(100)	34(100)	52(100)	45(100)
**GDM**				
Yes	0(0)	0(0)	0(0)	0(0)
No	5(100)	34(100)	52(100)	45(100)
**HDP**				
Yes	0(0)	0(0)	0(0)	0(0)
No	5(100)	34(100)	52(100)	45(100)
**Gestational thrombocytopenia**				
Yes	0(0)	0(0)	0(0)	0(0)
No	5(100)	34(100)	52(100)	45(100)
**FGR**				
Yes	0(0)	0(0)	0(0)	0(0)
No	5(100)	34(100)	52(100)	45(100)
**Macrosomia**				
Yes	0(0)	0(0)	0(0)	0(0)
No	5(100)	34(100)	52(100)	45(100)

ICP: intrahepatic cholestasis of pregnancy, **GDM:** gestational diabetes mellitus, **HDP:** hypertensive disorders of pregnancy, **FGR:** fetal growth restriction

Supplementary Table 2. Gestational weeks and cell ratio of all subjects

Sample	Stage	Gestational weeks	CD4+ Cytotoxic T	CD4+ Naive T	CD8+ cytotoxic T	CD8+ Naive T	DC	dnT	MAIT	Memory B cell	Monocyte	Naive B cell	NK	NK_CD56bright	NKT	other T	Plasmablast	Platelet	Proliferative T	Treg
control1_1	control	control	2.39%	17.12%	35.53%	14.85%	0.68%	7.95%	2.46%	2.54%	0.80%	2.50%	8.94%	1.29%	0.95%	0.04%	0.34%	0.23%	0.53%	0.87%
control1_2	control	control	3.01%	13.84%	38.28%	13.84%	0.36%	7.35%	2.37%	3.96%	0.31%	3.22%	9.35%	1.07%	0.87%	0.00%	0.23%	0.18%	0.46%	1.30%
control2_1	control	control	2.28%	19.94%	19.37%	28.47%	0.57%	14.19%	2.73%	2.00%	0.29%	1.06%	4.45%	1.14%	1.26%	0.00%	0.61%	0.24%	0.12%	1.26%
control2_2	control	control	2.56%	19.17%	15.44%	28.42%	0.59%	17.99%	3.64%	2.16%	0.49%	0.88%	4.52%	0.98%	1.18%	0.00%	0.29%	0.10%	0.29%	1.28%
control3_1	control	control	2.43%	16.13%	22.91%	16.88%	0.65%	13.65%	4.53%	2.66%	3.23%	5.28%	6.50%	0.51%	0.56%	1.54%	0.33%	0.05%	0.33%	1.82%
control3_2	control	control	2.51%	17.67%	24.00%	17.24%	0.72%	14.39%	5.65%	2.61%	1.50%	4.78%	5.84%	0.58%	0.72%	0.00%	0.19%	0.00%	0.39%	1.21%
control4_1	control	control	3.03%	13.64%	33.59%	17.55%	0.12%	9.61%	2.79%	1.95%	0.28%	1.72%	10.93%	1.32%	1.32%	0.00%	0.28%	0.04%	0.24%	1.60%
control4_2	control	control	3.59%	14.53%	28.44%	20.25%	0.28%	9.65%	3.98%	2.58%	0.06%	1.29%	10.49%	1.18%	1.07%	0.06%	0.17%	0.06%	0.34%	2.02%
control5_1	control	control	2.06%	14.06%	24.35%	20.40%	0.33%	17.91%	2.75%	2.81%	0.95%	2.84%	7.32%	0.49%	0.82%	0.00%	0.13%	0.26%	0.42%	2.09%
control5_2	control	control	2.06%	14.95%	21.92%	20.45%	0.23%	18.29%	3.30%	3.03%	0.83%	4.59%	6.65%	0.55%	0.83%	0.00%	0.00%	0.05%	0.23%	2.06%
NP150	6	early	3.15%	16.61%	28.23%	19.14%	0.79%	12.76%	2.27%	1.49%	4.28%	0.96%	5.68%	1.14%	1.22%	0.09%	0.35%	0.00%	0.61%	1.22%
NP152	6	early	1.34%	16.68%	11.12%	26.27%	0.96%	15.82%	4.31%	2.49%	4.99%	4.31%	6.33%	2.11%	0.29%	0.00%	0.77%	0.00%	0.48%	1.73%
NP78	6	early	2.21%	18.38%	21.21%	15.83%	0.96%	9.69%	3.50%	3.41%	1.92%	5.61%	11.37%	0.72%	1.01%	0.00%	0.91%	0.00%	1.10%	2.16%
NP87	6	early	2.00%	15.91%	21.69%	16.69%	1.11%	13.79%	5.23%	2.00%	2.22%	3.23%	11.35%	0.89%	1.11%	0.22%	0.11%	0.44%	0.89%	1.11%
NP115	7	early	1.17%	12.37%	25.91%	16.97%	1.04%	15.28%	4.99%	2.53%	2.98%	4.53%	7.12%	1.10%	0.84%	0.39%	1.36%	0.06%	0.26%	1.10%
NP120	7	early	2.51%	13.28%	20.87%	20.15%	0.53%	11.76%	6.27%	3.24%	1.32%	4.36%	11.56%	1.25%	0.59%	0.07%	0.33%	0.00%	0.33%	1.59%
NP124	7	early	1.70%	18.93%	25.33%	21.28%	0.26%	13.45%	1.83%	1.44%	1.83%	1.31%	8.88%	0.52%	0.39%	0.00%	0.52%	0.26%	0.52%	1.57%
NP76	7	early	2.30%	18.92%	19.81%	17.68%	0.51%	12.02%	1.75%	1.45%	6.26%	3.03%	10.52%	1.11%	0.60%	0.00%	0.47%	0.04%	1.36%	2.17%
NP103	8	early	2.46%	15.02%	30.48%	18.71%	0.44%	10.29%	3.30%	3.59%	3.55%	3.55%	4.14%	0.64%	0.59%	0.00%	0.10%	0.10%	0.84%	2.22%
NP111	8	early	2.75%	19.08%	27.62%	11.58%	0.67%	8.69%	5.35%	4.83%	5.20%	2.75%	6.98%	1.56%	1.04%	0.00%	0.89%	0.00%	0.30%	0.74%
NP117	8	early	2.95%	14.90%	29.60%	20.13%	0.40%	16.64%	2.01%	2.89%	0.20%	2.89%	4.09%	0.54%	1.41%	0.00%	0.13%	0.00%	0.07%	1.14%
NP130	8	early	2.01%	17.16%	34.74%	8.37%	1.04%	7.89%	3.25%	2.84%	3.74%	3.46%	10.24%	0.35%	1.11%	1.04%	0.69%	0.07%	0.42%	1.59%
NP75	8	early	3.47%	19.96%	24.83%	18.77%	1.26%	14.12%	4.73%	1.33%	2.22%	0.44%	5.40%	1.26%	0.30%	0.00%	0.52%	0.00%	0.22%	1.18%
NP54	9	early	2.13%	30.98%	23.11%	15.57%	1.15%	11.64%	0.98%	2.30%	1.48%	1.31%	4.59%	1.48%	0.66%	0.00%	0.66%	0.00%	0.16%	1.80%
NP65	9	early	1.68%	9.02%	29.64%	8.80%	0.56%	7.17%	3.87%	3.36%	3.64%	6.16%	16.13%	0.84%	0.73%	5.27%	0.22%	0.00%	2.07%	0.84%
NP74	9	early	2.84%	14.48%	32.25%	17.17%	0.56%	10.73%	5.85%	2.57%	0.89%	1.06%	6.47%	1.12%	1.51%	0.00%	0.24%	0.00%	0.21%	2.04%
NP79	9	early	2.27%	30.24%	17.03%	12.90%	0.83%	6.91%	8.46%	4.44%	0.93%	2.06%	11.15%	0.00%	0.31%	0.10%	0.31%	0.00%	2.06%	0.00%
NP104	10	early	2.55%	22.90%	24.36%	20.28%	1.02%	12.55%	4.60%	1.82%	0.44%	2.19%	3.21%	0.44%	0.88%	0.00%	0.15%	0.00%	0.88%	1.75%
NP108	10	early	3.11%	23.94%	27.98%	8.99%	0.64%	6.80%	6.29%	2.21%	1.67%	1.73%	12.80%	0.87%	0.67%	0.00%	0.39%	0.03%	0.42%	1.44%
NP6	10	early	1.80%	21.82%	19.73%	19.07%	0.85%	13.09%	7.12%	2.47%	1.71%	1.71%	6.26%	1.42%	1.04%	0.09%	0.00%	0.00%	0.38%	1.42%
NP85	10	early	4.56%	26.03%	23.68%	14.85%	0.29%	12.79%	3.97%	3.82%	1.76%	1.47%	3.09%	0.29%	0.44%	0.00%	0.44%	0.00%	0.74%	1.76%
NP107	11	early	3.05%	19.06%	30.43%	11.25%	0.95%	9.97%	4.32%	1.02%	3.49%	0.32%	10.93%	1.27%	1.08%	0.06%	0.64%	0.00%	1.08%	1.08%
NP123	11	early	2.18%	17.49%	25.45%	22.97%	0.15%	12.61%	5.33%	1.65%	1.20%	1.50%	5.33%	1.13%	0.75%	0.00%	0.38%	0.08%	0.15%	1.65%
NP56	11	early	2.08%	15.74%	26.94%	22.77%	0.72%	11.32%	3.97%	2.21%	1.37%	1.11%	6.70%	0.78%	1.37%	0.07%	0.78%	0.00%	0.85%	1.24%
NP57	11	early	4.79%	18.34%	20.60%	17.18%	0.82%	13.69%	1.98%	3.83%	1.23%	4.18%	9.17%	0.75%	0.62%	0.00%	1.03%	0.34%	0.21%	1.23%
NP94	11	early	2.43%	19.61%	35.33%	13.18%	0.54%	8.75%	4.11%	3.84%	0.81%	3.08%	4.70%	0.27%	0.54%	0.00%	0.97%	0.00%	0.49%	1.35%
NP110	12	early	1.61%	16.83%	26.58%	20.76%	0.91%	14.52%	4.98%	1.33%	0.28%	1.19%	4.70%	0.84%	0.42%	0.00%	1.68%	0.00%	0.35%	3.02%
NP121	12	early	3.35%	22.04%	25.82%	17.16%	0.36%	15.93%	2.47%	3.85%	1.09%	1.53%	2.69%	0.36%	0.80%	0.07%	0.22%	0.07%	0.73%	1.45%
NP49	12	early	3.01%	15.88%	23.63%	16.42%	1.46%	14.05%	7.48%	1.09%	2.92%	3.10%	7.66%	1.00%	0.82%	0.00%	0.09%	0.00%	0.27%	1.09%
NP92	12	early	4.83%	23.57%	22.69%	16.92%	0.65%	10.27%	3.86%	3.44%	0.84%	3.67%	4.60%	1.12%	0.74%	0.05%	0.70%	0.00%	0.46%	1.58%
NP119	13	early	3.10%	18.43%	29.59%	18.55%	0.31%	13.32%	2.12%	2.87%	0.28%	3.77%	3.22%	0.79%	1.34%	0.00%	0.20%	0.04%	0.43%	1.65%
NP52	13	early	2.01%	18.25%	20.55%	24.71%	0.57%	15.80%	3.16%	0.72%	2.44%	3.02%	4.74%	0.86%	1.01%	0.00%	0.57%	0.00%	0.72%	0.86%
NP77	13	early	3.40%	21.69%	30.48%	16.94%	0.44%	7.02%	6.65%	4.00%	0.42%	2.12%	3.45%	0.53%	0.75%	0.04%	0.18%	0.13%	0.40%	1.37%
NP80	13	early	2.09%	19.04%	40.14%	9.50%	0.44%	9.14%	1.85%	2.54%	1.85%	2.78%	5.72%	0.56%	0.89%	0.00%	0.44%	0.08%	0.68%	2.25%
NP126	14	mid	5.49%	20.91%	31.78%	7.83%	0.58%	11.33%	3.04%	2.69%	0.35%	0.70%	5.84%	3.86%	1.75%	0.00%	1.05%	0.23%	0.70%	1.87%
NP21	14	mid	2.24%	18.16%	20.85%	21.30%	0.22%	12.11%	1.79%	1.35%	4.93%	4.48%	8.30%	1.12%	0.45%	0.00%	0.90%	0.00%	0.67%	1.12%
NP70	14	mid	2.32%	16.86%	30.74%	14.96%	0.98%	10.88%	3.56%	3.61%	3.40%	3.04%	2.94%	0.83%	1.03%	3.20%	0.15%	0.15%	0.41%	0.93%
NP109	15	mid	1.13%	17.80%	21.60%	20.20%	0.67%	14.80%	2.47%	3.93%	1.27%	6.53%	5.33%	0.27%	0.60%	0.00%	0.60%	0.13%	0.47%	2.20%
NP116	15	mid	2.37%	16.70%	23.52%	21.74%	0.89%	15.61%	1.98%	3.06%	0.49%	2.37%	8.40%	0.30%	0.40%	0.00%	0.49%	0.20%	0.30%	1.19%
NP153	15	mid	3.05%	11.07%	30.73%	11.45%	1.15%	5.34%	3.44%	1.72%	16.03%	1.72%	8.59%	0.76%	1.34%	0.38%	0.19%	0.19%	2.10%	0.76%
NP154	15	mid	1.63%	15.53%	29.86%	19.45%	0.36%	9.69%	1.08%	1.20%	3.55%	0.96%	9.51%	0.60%	0.90%	1.69%	0.42%	0.06%	2.41%	1.08%
NP36	15	mid	1.15%	12.01%	24.52%	21.58%	0.38%	17.24%	1.66%	1.28%	7.28%	1.02%	6.51%	1.40%	0.38%	0.26%	1.02%	0.00%	0.77%	1.53%
NP24	16	mid	1.43%	22.81%	10.90%	25.68%	0.14%	16.50%	2.73%	0.43%	5.16%	1.29%	7.75%	1.29%	1.43%	0.00%	0.14%	0.14%	0.29%	1.87%
NP55	16	mid	3.87%	13.80%	32.21%	14.73%	0.70%	8.25%	5.45%	5.87%	0.33%	4.10%	6.57%	0.56%	1.49%	0.00%	0.42%	0.05%	0.61%	0.98%
NP122	17	mid	2.64%	24.09%	20.01%	18.48%	0.41%	13.74%	4.13%	2.72%	0.87%	1.53%	4.46%	1.16%	0.99%	0.00%	1.11%	0.00%	1.65%	2.02%
NP82	17	mid	2.48%	12.88%	22.38%	25.14%	0.55%	14.81%	7.51%	2.13%	1.03%	4.89%	2.96%	0.62%	0.62%	0.00%	0.48%	0.07%	0.28%	1.17%
NP12	18	mid	3.85%	19.46%	19.27%	25.82%	0.96%	12.14%	0.77%	2.70%	0.58%	1.35%	7.13%	1.93%	0.19%	0.00%	0.00%	0.58%	0.39%	2.89%
NP142	18	mid	4.19%	10.03%	33.50%	10.79%	0.76%	8.50%	3.05%	1.78%	6.22%	1.90%	9.90%	1.52%	0.76%	4.19%	1.02%	0.00%	0.76%	1.14%
NP145	18	mid	1.22%	17.44%	15.68%	24.33%	0.84%	17.06%	1.15%	1.07%	3.44%	3.52%	8.57%	1.07%	0.46%	2.22%	0.46%	0.00%	0.31%	1.15%
NP146	18	mid	2.73%	17.99%	24.86%	22.90%	0.65%	13.96%	2.40%	0.22%	4.47%	0.76%	3.60%	1.42%	1.09%	0.00%	0.87%	0.33%	1.09%	0.65%
NP64	18	mid	1.67%	17.73%	25.64%	19.80%	1.27%	9.06%	2.77%	4.27%	2.31%	3.81%	6.12%	0.52%	1.27%	0.06%	1.21%	0.12%	0.98%	1.39%
NP100	19	mid	0.76%	15.37%	26.94%	20.70%	2.59%	14.61%	0.61%	0.00%	5.18%	0.76%	6.85%	1.22%	0.76%	0.00%	1.52%	0.00%	0.30%	1.83%
NP102	19	mid	1.70%	18.93%	25.33%	21.28%	0.26%	13.45%	1.83%	1.44%	1.83%	1.31%	8.88%	0.52%	0.39%	0.00%	0.52%	0.26%	0.52%	1.57%
NP131	19	mid	1.97%	10.59%	40.11%	13.12%	0.28%	9.93%	4.87%	1.97%	5.72%	1.12%	5.53%	0.19%	1.12%	0.94%	0.84%	0.09%	0.19%	1.41%
NP156	19	mid	1.55%	14.16%	35.84%	16.69%	0.65%	14.32%	1.55%	0.49%	2.54%	0.82%	5.56%	0.90%	0.74%	0.00%	0.65%	0.16%	0.90%	2.45%
NP125	20	mid	2.07%	18.59%	27.42%	11.75%	0.38%	8.83%	5.15%	2.30%	6.68%	0.84%	11.14%	0.61%	0.77%	0.00%	0.54%	0.31%	0.69%	1.92%
NP138	20	mid	3.98%	16.90%	28.35%	10.84%	0.80%	10.13%	7.19%	3.17%	6.58%	1.18%	6.77%	1.23%	0.85%	0.05%	0.47%	0.00%	0.43%	1.09%
NP45	20	mid	1.52%	26.03%	27.98%	16.27%	0.87%	5.86%	5.42%	1.08%	3.04%	1.30%	6.94%	0.43%	0.87%	0.00%	0.43%	0.22%	0.87%	0.87%
NP53	20	mid	3.59%	11.95%	35.61%	12.11%	0.82%	11.44%	6.57%	3.49%	1.80%	4.05%	5.54%	0.21%	0.77%	0.00%	0.26%	0.10%	0.41%	1.28%
NP147	21	mid	1.52%	13.52%	27.15%	24.51%	0.51%	18.76%	4.06%	0.90%	2.14%	0.85%	1.07%	0.39%	1.30%	0.11%	0.62%	0.23%	0.51%	1.86%
NP148	21	mid	2.53%	18.62%	19.54%	28.58%	0.69%	12.18%	3.68%	2.30%	0.77%	1.76%	5.59%	0.69%	0.46%	0.00%	0.84%	0.08%	0.61%	1.07%
NP149	21	mid	2.86%	13.79%	32.91%	20.53%	0.18%	9.21%	0.97%	2.69%	0.53%	2.47%	7.27%	1.81%	0.48%	0.00%	0.75%	0.22%	1.85%	1.50%
NP28	21	mid	2.17%	17.83%	32.17%	17.83%	0.50%	13.83%	1.50%	2.33%	0.50%	2.33%	5.17%	0.17%	1.00%	0.00%	0.67%	0.00%	1.00%	1.00%
NP101	22	mid	2.26%	18.38%	25.64%	19.91%	0.55%	13.12%	4.62%	2.22%	4.06%	1.71%	3.60%	0.65%	0.28%	0.00%	0.46%	0.05%	0.83%	1.66%
NP14	22	mid	2.90%	10.41%	38.52%	16.85%	0.86%	11.48%	3.00%	2.68%	1.50%	1.82%	5.58%	0.97%	0.97%	0.00%	0.00%	0.43%	0.86%	1.18%
NP132	23	mid	1.10%	11.51%	34.99%	10.22%	1.47%	16.67%	1.93%	2.12%	5.80%	2.58%	4.51%	2.30%	1.38%	0.09%	1.29%	0.18%	1.20%	0.64%
NP26	23	mid	2.32%	16.49%	13.49%	23.57%	0.27%	18.39%	1.36%	1.23%	3.00%	1.77%	13.76%	0.95%	1.23%	0.00%	0.27%	0.00%	0.41%	1.50%
NP47	23	mid	1.79%	15.65%	13.50%	35.36%	0.72%	16.73%	3.70%	1.55%	1.19%	1.43%	5.26%	0.48%	0.72%	0.00%	0.12%	0.12%	0.36%	1.31%
NP50	23	mid	1.62%	17.90%	22.07%	17.77%	1.62%	11.71%	1.48%	1.08%	6.06%	1.62%	10.50%	0.67%	1.21%	0.00%	1.21%	0.27%	0.81%	2.42%
NP99	23	mid	2.06%	21.90%	11.81%	20.99%	0.57%	16.06%	1.38%	0.92%	9.17%	1.72%	4.93%	1.61%	1.03%	0.00%	1.61%	0.57%	0.46%	3.21%
NP106	24	mid	1.87%	15.16%	18.59%	24.79%	0.24%	19.92%	1.56%	1.93%	0.72%	3.79%	7.04%	0.90%	0.36%	0.00%	0.66%	0.06%	0.96%	1.44%
NP58	24	mid	1.66%	13.82%	28.30%	19.57%	0.39%	10.45%	2.21%	2.99%	1.77%	3.54%	9.23%	1.00%	0.72%	0.00%	1.22%	0.06%	0.83%	2.27%
NP72	24	mid	2.15%	10.24%	23.11%	22.15%	0.35%	18.69%	5.61%	1.87%	1.80%	3.11%	6.37%	1.80%	0.42%	0.00%	0.48%	0.21%	0.55%	1.11%
NP96	24	mid	6.45%	28.60%	24.30%	11.83%	1.29%	10.54%	1.72%	0.65%	4.95%	1.29%	4.52%	0.22%	0.22%	0.00%	0.86%	0.00%	0.43%	2.15%
NP31	25	mid	2.99%	24.31%	29.42%	19.19%	0.21%	10.23%	2.13%	0.64%	0.85%	1.49%	3.20%	0.64%	0.64%	0.00%	0.64%	0.00%	1.49%	1.92%
NP61	25	mid	3.23%	24.18%	19.20%	19.53%	0.49%	12.04%	5.69%	3.56%	1.81%	2.19%	3.99%	0.98%	0.88%	0.00%	0.55%	0.16%	0.55%	0.98%
NP88	25	mid	2.56%	29.14%	21.68%	15.15%	1.17%	9.79%	0.47%	3.03%	3.73%	1.86%	3.96%	0.23%	0.00%	0.00%	1.17%	0.93%	3.26%	1.86%
NP97	25	mid	2.02%	29.29%	16.16%	16.92%	0.00%	16.41%	2.02%	0.00%	5.30%	0.25%	2.27%	0.51%	1.52%	0.00%	1.77%	0.00%	1.01%	4.55%
NP105	26	mid	2.34%	18.00%	23.15%	23.64%	0.86%	10.48%	4.81%	2.87%	2.97%	2.44%	5.28%	0.53%	0.56%	0.00%	0.30%	0.07%	0.53%	1.19%
NP30	26	mid	1.15%	13.54%	30.43%	20.36%	0.10%	15.42%	1.89%	3.46%	0.63%	3.36%	5.46%	0.31%	1.47%	0.10%	0.52%	0.00%	0.52%	1.26%
NP86	26	mid	1.89%	10.80%	60.99%	6.46%	0.08%	5.20%	0.87%	2.21%	1.89%	5.67%	2.52%	0.08%	0.32%	0.24%	0.39%	0.00%	0.39%	0.00%
NP89	26	mid	3.40%	19.96%	30.77%	12.39%	0.56%	11.93%	4.98%	2.95%	1.44%	2.07%	6.11%	0.74%	1.02%	0.00%	0.39%	0.07%	0.46%	0.77%
NP128	27	mid	2.13%	15.30%	19.75%	23.30%	0.68%	12.03%	6.27%	5.81%	1.82%	5.95%	3.13%	0.64%	0.45%	0.23%	0.32%	0.09%	0.09%	2.00%
NP139	27	mid	3.86%	16.77%	15.13%	24.04%	0.74%	15.88%	5.79%	1.93%	1.04%	1.78%	9.94%	0.45%	0.15%	0.30%	0.45%	0.00%	1.19%	0.59%
NP39	27	mid	1.87%	13.14%	29.93%	18.09%	0.24%	15.49%	3.08%	2.11%	0.81%	1.22%	8.92%	0.73%	1.46%	0.08%	0.57%	0.24%	0.81%	1.22%
NP4	27	mid	1.85%	16.96%	17.06%	29.82%	0.58%	16.18%	2.53%	2.05%	1.56%	4.19%	4.48%	0.58%	0.49%	0.00%	0.19%	0.00%	0.39%	1.07%
NP118	28	late	2.35%	14.04%	43.58%	11.69%	0.65%	5.50%	3.45%	2.75%	3.00%	1.50%	6.10%	1.30%	1.35%	0.05%	0.70%	0.40%	0.50%	1.10%
NP140	28	late	2.14%	13.34%	29.26%	23.52%	0.77%	15.40%	5.90%	1.80%	1.71%	0.86%	0.86%	0.77%	0.77%	0.00%	0.34%	0.00%	1.45%	1.11%
NP129	29	late	1.81%	13.48%	27.86%	16.02%	0.41%	15.37%	8.05%	1.31%	3.04%	1.64%	7.48%	0.66%	0.66%	0.00%	0.41%	0.16%	0.33%	1.31%
NP136	29	late	1.14%	10.91%	27.52%	16.37%	1.80%	13.25%	3.54%	3.30%	11.75%	1.56%	5.70%	0.18%	0.36%	0.30%	0.60%	0.42%	0.90%	0.42%
NP137	29	late	1.63%	15.07%	23.61%	19.75%	0.30%	21.31%	5.12%	2.60%	0.82%	1.86%	3.27%	0.67%	0.82%	0.00%	0.45%	0.52%	1.04%	1.19%
NP5	29	late	1.68%	16.97%	29.72%	18.17%	0.72%	9.75%	3.73%	3.25%	3.85%	4.09%	3.01%	0.84%	0.24%	0.00%	0.72%	0.36%	1.44%	1.44%
NP90	29	late	3.12%	24.97%	15.61%	19.81%	0.97%	11.63%	4.09%	1.94%	2.15%	0.32%	8.18%	1.40%	0.75%	0.22%	0.75%	0.43%	1.72%	1.94%
NP17	30	late	1.81%	11.33%	25.98%	17.82%	0.00%	14.95%	4.98%	1.36%	7.10%	3.78%	2.57%	1.21%	0.45%	4.08%	0.30%	0.00%	0.91%	1.36%
NP20	30	late	2.21%	15.35%	42.90%	11.67%	0.42%	10.09%	3.89%	0.53%	3.58%	1.37%	4.52%	0.84%	0.74%	0.00%	0.32%	0.21%	0.32%	1.05%
NP34	30	late	2.22%	18.95%	20.16%	25.60%	0.20%	16.73%	1.61%	1.41%	2.82%	1.21%	4.03%	0.20%	1.01%	0.00%	0.20%	0.20%	1.21%	2.22%
NP73	30	late	2.66%	20.67%	18.01%	18.92%	1.37%	12.84%	2.74%	1.29%	4.33%	1.98%	10.94%	0.23%	0.30%	0.00%	0.30%	0.15%	0.76%	2.51%
NP81	30	late	2.05%	17.05%	23.75%	19.70%	0.15%	17.65%	2.10%	1.35%	1.65%	1.60%	8.20%	0.70%	0.50%	0.00%	0.30%	0.30%	0.95%	2.00%
NP113	31	late	1.91%	18.41%	23.95%	16.54%	0.49%	16.42%	3.42%	3.10%	0.37%	9.49%	2.32%	0.65%	0.90%	0.00%	0.81%	0.00%	0.16%	1.06%
NP135	31	late	1.90%	13.58%	24.09%	14.45%	2.48%	12.99%	5.55%	4.96%	6.42%	1.31%	6.42%	0.00%	0.58%	0.58%	0.73%	0.15%	3.36%	0.44%
NP25	31	late	1.93%	17.24%	31.58%	15.63%	0.54%	11.46%	1.39%	1.82%	5.14%	1.61%	4.07%	0.75%	1.82%	0.75%	1.50%	0.32%	1.18%	1.28%
NP38	31	late	3.77%	15.53%	33.57%	18.31%	0.18%	8.98%	2.42%	1.71%	4.58%	2.96%	4.76%	0.54%	0.27%	0.00%	0.72%	0.00%	0.45%	1.26%
NP59	32	late	2.37%	24.54%	19.27%	23.55%	0.08%	14.60%	3.98%	2.14%	1.99%	1.68%	1.99%	0.69%	0.69%	0.00%	0.54%	0.08%	0.69%	1.15%
NP60	32	late	2.09%	16.54%	19.77%	20.34%	0.38%	13.88%	1.90%	3.80%	2.85%	4.37%	11.03%	0.57%	0.00%	0.00%	0.19%	0.00%	0.19%	2.09%
NP71	32	late	3.59%	20.27%	18.21%	20.84%	0.67%	10.21%	8.62%	6.28%	2.40%	1.29%	3.88%	0.53%	0.62%	0.00%	0.57%	0.05%	1.01%	0.96%
NP19	33	late	3.09%	16.67%	19.14%	16.67%	1.23%	11.11%	5.86%	3.70%	6.48%	2.78%	8.33%	0.62%	0.31%	0.31%	0.31%	0.62%	1.54%	1.23%
NP27	33	late	1.33%	12.09%	29.41%	25.41%	0.41%	16.60%	0.72%	1.43%	3.59%	0.92%	3.59%	0.82%	1.23%	0.10%	0.41%	0.51%	0.61%	0.82%
NP63	33	late	3.22%	18.56%	23.71%	22.00%	0.52%	13.52%	2.34%	2.39%	1.35%	1.46%	5.36%	0.57%	0.52%	1.20%	0.62%	0.26%	0.99%	1.40%
NP83	33	late	3.58%	15.69%	17.59%	26.93%	0.58%	12.63%	8.25%	2.92%	2.34%	1.53%	3.50%	0.66%	0.36%	0.15%	0.51%	0.07%	0.88%	1.82%
NP37	34	late	2.13%	13.55%	31.09%	13.37%	0.00%	8.86%	1.24%	1.51%	3.99%	4.07%	7.97%	0.62%	0.71%	8.59%	0.18%	0.00%	1.15%	0.97%
NP43	34	late	1.70%	16.27%	27.72%	12.58%	0.38%	8.23%	3.22%	2.74%	5.87%	3.60%	6.43%	0.66%	0.95%	5.11%	0.95%	0.28%	1.51%	1.80%
NP8	34	late	2.37%	14.88%	21.74%	18.54%	0.19%	11.80%	3.59%	2.76%	5.32%	4.62%	9.17%	1.28%	0.71%	0.19%	0.19%	0.00%	0.58%	2.05%
NP112	35	late	2.63%	17.94%	20.26%	16.27%	0.80%	12.44%	7.26%	1.99%	3.59%	1.20%	10.29%	1.83%	0.56%	0.00%	0.32%	0.56%	0.96%	1.12%
NP127	35	late	1.44%	13.83%	33.35%	19.14%	0.57%	11.24%	2.97%	3.78%	1.10%	2.68%	4.93%	0.67%	0.24%	0.00%	0.77%	0.43%	0.72%	2.15%
NP40	35	late	3.90%	17.14%	25.97%	14.81%	0.26%	10.39%	2.86%	3.38%	4.42%	2.60%	10.13%	0.26%	0.26%	0.00%	0.00%	0.00%	2.34%	1.30%
NP44	35	late	2.73%	14.77%	30.91%	17.95%	0.23%	12.50%	2.05%	2.27%	3.41%	1.36%	6.59%	0.45%	0.45%	0.00%	0.91%	0.00%	2.27%	1.14%
NP32	36	late	0.44%	10.19%	33.92%	10.63%	0.53%	14.59%	0.53%	1.23%	5.71%	6.41%	10.98%	0.79%	0.53%	0.53%	0.88%	0.26%	0.26%	1.58%
NP35	36	late	1.45%	12.40%	29.34%	15.29%	0.41%	10.74%	2.07%	2.89%	4.55%	0.00%	15.50%	0.21%	1.24%	0.21%	0.62%	0.83%	1.03%	1.24%
NP48	36	late	1.76%	13.56%	22.54%	17.96%	0.18%	14.08%	4.40%	1.41%	4.23%	2.82%	11.97%	0.70%	0.88%	0.35%	0.18%	0.18%	0.70%	2.11%
NP66	37	late	1.45%	13.41%	47.74%	11.41%	0.45%	7.52%	2.90%	1.81%	1.72%	1.99%	4.44%	0.45%	2.54%	0.09%	0.54%	0.00%	0.82%	0.72%
NP69	37	late	0.28%	17.20%	17.34%	30.65%	0.55%	15.95%	5.55%	0.83%	3.47%	0.42%	3.88%	1.11%	0.42%	0.00%	0.42%	0.14%	0.55%	1.25%
NP7	37	late	3.46%	14.90%	15.33%	22.68%	0.22%	14.04%	4.10%	2.81%	4.54%	0.43%	12.31%	0.65%	0.43%	0.00%	0.22%	0.00%	1.30%	2.59%
NP9	37	late	1.77%	16.59%	26.11%	25.66%	0.00%	17.48%	2.88%	0.88%	2.88%	1.11%	2.21%	0.22%	0.44%	0.00%	0.00%	0.66%	0.22%	0.88%
NP155	38	late	2.80%	18.53%	38.02%	11.89%	0.26%	12.06%	1.22%	0.70%	2.36%	0.44%	6.38%	0.79%	1.40%	0.00%	0.17%	0.61%	0.61%	1.75%
NP41	38	late	1.56%	21.25%	36.06%	14.62%	0.78%	9.36%	0.39%	0.39%	4.48%	0.58%	5.07%	0.19%	0.58%	0.00%	0.00%	0.19%	1.56%	2.92%
NP10	39	late	2.93%	16.41%	31.45%	19.73%	1.56%	9.18%	3.71%	1.17%	1.37%	0.59%	8.40%	0.59%	1.17%	0.00%	0.00%	0.00%	0.78%	0.98%
NP114	39	late	3.59%	21.16%	29.06%	17.35%	0.53%	11.36%	2.66%	2.53%	1.55%	1.73%	3.37%	0.62%	0.84%	0.00%	0.44%	0.22%	0.80%	2.17%
NP159	39	late	3.10%	20.89%	21.92%	16.38%	0.22%	10.33%	5.76%	1.99%	1.62%	0.74%	12.03%	1.11%	0.52%	0.00%	0.15%	0.15%	0.96%	2.14%
NP67	39	late	2.77%	13.61%	24.33%	21.96%	0.35%	16.60%	1.84%	3.47%	1.54%	4.26%	3.73%	0.97%	0.83%	0.53%	0.57%	0.40%	0.57%	1.67%
NP91	39	late	2.93%	19.17%	33.28%	15.45%	0.59%	10.83%	6.40%	1.90%	1.75%	1.34%	2.75%	0.94%	0.50%	0.03%	0.16%	0.25%	0.50%	1.22%
NP158	40	late	2.07%	17.97%	28.57%	22.00%	0.12%	14.29%	1.73%	1.15%	1.61%	2.19%	2.30%	0.58%	0.46%	0.00%	0.69%	0.12%	1.73%	2.42%

Supplementary Table 3A. The number of single cells and pseudocells in the training dataset and the independent testing dataset, with the number of features selected by Lasso

Cell type	Total single cells(N)	Training dataset		Independent testing dataset		Selected Features by Lasso (N)
		Single cells (N)	Pseudocells (N)	Single cells (N)	Pseudocells (N)	
CD8+ cytotoxic T	47043	32930	3234	14113	131	1615
CD8+ Naive T	31258	21880	2131	9378	131	1335
CD4+ Naive T	30180	21126	2055	9054	131	1284
dnT	21240	14868	1427	6372	131	1044
NK	10875	7612	710	3263	131	600
MAIT	6775	4742	434	2033	131	447
Monocyte	4490	3143	283	1347	131	309
Memory B cell	4460	3122	290	1338	129	325
Naive B cell	4426	3097	295	1328	129	275
CD4+ Cytotoxic T	4348	3043	271	1305	131	286
Treg	2587	1810	171	777	129	185
NK_CD56bright	1431	995	125	427	120	139
NKT	1372	957	130	411	123	132
Proliferative T	1268	884	131	380	126	147

Supplementary Table 3B. RMSE values calculated by 14 cell-type-specific models with the optimal hyperparameters determined by the 5-fold cross validation in the training dataset

Cell type	Optimal hyperparameter*				RMSE	
	n_estimators	min_samples_split	min_samples_leaf	max_depth	mean	std
CD8+ cytotoxic T	100	2	2	190	14.730	0.407
CD8+ Naive T	100	2	1	65	14.871	0.327
CD4+ Naive T	100	2	1	65	15.293	0.372
dnT	70	2	2	195	17.097	0.781
NK	100	2	1	65	15.975	0.328
MAIT	100	2	2	190	20.134	1.826
Monocyte	100	2	1	15	21.252	1.495
Memory B cell	80	5	1	165	23.483	1.612
Naive B cell	55	5	2	30	25.051	1.414
CD4+ Cytotoxic T	100	2	1	65	20.875	0.728
Treg	85	5	2	175	25.349	0.996
NK_CD56bright	85	5	2	175	25.871	3.671
NKT	30	2	2	160	26.990	3.161
Proliferative T	60	2	4	20	33.395	2.372

*hyperparameter space:
n_estimators = [10, 20, 30, …, 100],
max_depth = [10, 15, 20, …, 205],
min_samples_split = [2, 5, 10],
min_samples_leaf = [1, 2, 4],
All other hyperparameters were configured with default values in the scikit-learn libaray.
